# Differential Strategies of Ectomycorrhizal Development between *Suillus luteus* and *Pinus massoniana* in Response to Nutrient Changes

**DOI:** 10.3390/jof10080587

**Published:** 2024-08-19

**Authors:** Xueyu Pan, Junfeng Liang, Jinhua Zhang, Yan Zhao, Mingjie Chen

**Affiliations:** 1 Institute of Edible Fungi, Shanghai Academy of Agricultural Sciences, Shanghai 201403, China; panxueyu@saas.sh.cn; 2 Research Institute of Tropical Forestry, Chinese Academy of Forestry, Guangzhou 510520, China; jfliang2000@163.com (J.L.); jhzhang0111@163.com (J.Z.); 3 Shanghai Key Laboratory of Agricultural Genetics and Breeding, Shanghai Academy of Agricultural Sciences, Shanghai 201106, China

**Keywords:** mutualism, colonization, RNA-seq, edible mushrooms, fungi

## Abstract

Ectomycorrhizal fungi employ different strategies for mycelial growth and host colonization under varying nutrient conditions. However, key genes associated with mycorrhizal interaction should be influenced solely by the inoculation treatment and not by nutrient variations. To utilize subtle nutrient differences and rapidly screen for key genes related to the interaction between *Suillus luteus* and *Pinus massoniana*, we performed an inoculation experiment using culture bottles containing high- and low-nutrient media. Interestingly, *S. luteus* LS88 promoted the growth of *P. massoniana* seedlings without mature ectomycorrhiza, and the impact of LS88 inoculation on *P. massoniana* roots was greater than that of nutrient changes. In this study, the resequenced genome of the LS88 strain was utilized for transcriptome analysis of the strain. The analysis indicated that a unique gene encoding glutathione S-transferase (GST) in LS88 is likely involved in colonizing *P. massoniana* roots. In this study, the GST gene expression was independent of nutrient levels. It was probably induced by *P. massoniana* and could be used as a marker for *S. luteus* colonization degree.

## 1. Introduction

Mycorrhizal fungi play an important role in the forest microbial community by forming mutualistic symbiotic relationships with most plant species on Earth, thereby influencing the growth and development of host plants [1]. Since the discovery of mycorrhizae, extensive research has been conducted on their functions, ecology, and distribution [2]. The ectomycorrhizal (ECM) fungal group includes many edible mushrooms that not only enhance host plant growth but also have significant economic benefits. In recent years, significant progress has been made in understanding the mechanisms of ECM development, particularly in the *Laccaria bicolor*–*Populus* spp. mycorrhizal system [3]. It has been demonstrated that this fungus can influence the defense responses and root development of poplar plants.

Mycorrhizal fungi can assist plants in nutrient and water absorption in various ways [4] and play pivotal roles in nutrient cycling in ecosystems [5]. ECM fungi can drive the activation and transport of nitrogen [6] and regulate carbon accumulation [7] in the soil. During symbiosis, many mycorrhizal fungi can secrete signaling proteins that impact their plant hosts [8]. For example, the ECM fungus *L. bicolor* produces the MiSSP7 protein, which stabilizes the inhibition of the jasmonic acid signaling repressor PtJAZ67 in poplar trees [9]. ECM fungi can also influence the root development of the host, thereby forming mature mycorrhizal structures with the host root system [10]. However, the impact of nutrient levels on the recognition of ECM fungi by the host is still not fully understood. Nonetheless, analyzing the transcriptomic changes in ECM fungi and their hosts under different nutrient levels may reveal genes involved in ECM establishment.

As ECM fungi in Pinaceae plants, *Suillus* fungi have accumulated a substantial body of literature and are crucial for studying ECM interactions in coniferous trees [11]. Utilizing metatranscriptomics to study the interaction between pine trees and *Suillus* fungi shows great promise in identifying genes involved in these mutualistic processes [12]. This approach enables rapid identification of functional genes within the samples and provides a new insight into the ECM interaction mechanisms [13]. A recent study showed that pine trees can form ECM associations regardless of the addition of extra iron after inoculation with *Suillus* strains [14]. This indicates that the expression of key genes involved in the *Suillus-Pinus* ECM interaction may not be significantly affected by minor nutrient changes.

As a native tree species in southern China, *Pinus massoniana* is a typical ECM plant. The edible ECM fungus *Suillus luteus* is an excellent mycorrhizal partner for *P. massoniana*, and the two have a high rate of mycorrhizal synthesis [15,16]. For *P. massoniana*, inoculation with *S. luteus* not only helps to expand the root distribution range but also promotes biomass accumulation and the uptake of trace elements by seedlings [17,18,19]. Under different cultivation conditions, *S. luteus* can promote the growth of *P. massoniana* in various ways: under phosphorus deficiency, *S. luteus* enhances the ability of *P. massoniana* to absorb phosphorus and potassium [20], and inoculation with *S. luteus* also regulates the soil microbiome of *P. massoniana*, assisting in resistance against heavy metal stress [21]. Furthermore, complete genome information for *S. luteus* has been published [22], providing a foundation for genomic studies of this fungus.

In a previous study, we synthesized *S. luteus*–*P. massoniana* mycorrhizae and confirmed the stable growth-promoting effect of the *S. luteus* strain LS88 on *P. massoniana* seedlings [23]. Here, we hypothesize that creating slight nutrient differences will help in rapidly identifying genes whose expression trends are influenced by the inoculation treatment and not by nutrient changes. We conducted this study by germinating sterile *P. massoniana* seedlings and inoculating them with *S. luteus* strain LS88 in culture bottles containing different nutrients. High-quality samples were collected for transcriptomic analysis. By searching for genes that exhibit significant expression in the inoculated roots at both nutrient levels, our study identified one *S. luteus* gene linked to host recognition and three *S. luteus* genes involved in mycorrhizal association. Moreover, the results revealed that reducing nutrient levels appropriately enhanced the ability of LS88 to colonize *P. massoniana* roots. Additionally, inoculation with LS88 had a greater impact on *P. massoniana* growth than increasing the nutrient content in the growth medium.

## 2. Materials and Methods

### 2.1. Fungal Materials and Growing Conditions

The *S. luteus* strain LS88 originated from the Research Institute of Tropical Forestry, Chinese Academy of Forestry, Guangzhou, P.R. People’s Republic of China, and was isolated from the fruiting body of *S. luteus* under *P. massoniana* [23]. Strain LS88 was precultured in the dark at 24 °C on adjusted potato dextrose agar media [23].

For genome resequencing, after 30 days of cultivation, LS88 mycelia were punched with a 1 cm diameter perforator to obtain mycelial plugs. Ten mycelial plugs were added to a 500 mL flask containing 300 mL of adjusted potato dextrose media, and the culture was incubated in a shaker at 150 rpm and 24 °C in the dark. After 60 days, the mycelia were collected and filtered through a sterile sieve (90 mesh, diameter 6 cm) to remove the media. The mycelia were frozen in liquid nitrogen and stored at −80 °C for subsequent genome resequencing and transcriptome analysis.

### 2.2. Plant Materials and Growing Conditions

*P. massoniana* seeds were supplied by the Xinyi Forestry Research Institute, Guangdong Province, China. The most recently obtained batch of *P. massoniana* seeds was selected and stored in a sealed Ziplock bag at 4 °C for preservation. Before germination, plump *P. massoniana* seeds were carefully selected on a clean bench. The episperm of the *P. massoniana* seeds was then removed, and the surfaces were promptly disinfected to ensure proper hygiene and minimize the potential for contamination.

To produce sterile seedlings for inoculation, we disinfected the surface of the seeds with a 0.1% mercuric chloride solution. The main steps for surface disinfection of *P. massoniana* seeds were as follows: (a) The seeds were rinsed three times with sterile water. (b) The seeds were soaked in a 0.1% mercuric chloride solution for 7 min. (c) After soaking, the seeds were rinsed three times with sterile water and soaked in sterile water for 5 min; this step was repeated three times. (d) Following rinsing and soaking, the seeds were rinsed three times with sterile water. (e) The sterile seeds were transferred to a germination media tube using sterilized tweezers. Sterility was ensured by performing these steps on a sterile table using a sterile sieve (90 mesh, diameter 6 cm). All tools involved, such as tweezers and beakers, were autoclaved.

Adjusted Murashige and Skoog media [24] was used to germinate sterile *P. massoniana* seedlings; media contained 5.8428 mM of sucrose (C_12_H_22_O_11_), 1.8738 mM of potassium phosphate monobasic (H_2_KO_4_P), 16.7903 mM of potassium nitrate (KNO_3_), 991.1696 µM of calcium chloride (CaCl_2_), 25 nM of boric acid (BH_3_O_3_), 13.3168 µM of zinc sulfate (O_4_SZn), 39.1579 nM of copper sulfate (CuO_4_S), 25 µM of ferrous sulfate heptahydrate (FeH_14_O_11_S), 6.6605 µM of glycine (C_2_H_5_NO_2_), 607.8584 nM of pyridoxine hydrochloride (C_8_H_11_NO_3_·HCl), 27.7356 µM of EDTA disodium salt (C_10_H_14_N_2_Na_2_O_8_), 5.1537 mM of ammonium nitrate (NH_4_NO_3_), 768.4639 µM of magnesium sulfate (MgO_4_S), 1.25 µM of potassium iodide (IK), 36.9205 µM of manganese sulphate (MnO_4_S), 303.5159 nM of sodium molybdate (MoNa_2_O_4_), 48.1362 nM of cobalt chloride (Cl_2_Co), 138.7655 µM of inositol (C_6_H_12_O_6_), 74.1246 nM of thiamine hydrochloride (C_12_H_18_C_l2_N_4_OS), 1.0236 µM of niacinamide (C_6_H_6_N_2_O) and 1.2 g of phytagel per liter. The media was prepared by boiling until it was uniform in texture. The solution was then dispensed into 1.8 cm inner-diameter test tubes, with each tube containing 5 mL of the media. The test tubes were sealed with breathable plugs and sterilized at 121 °C for 30 min in an autoclave. After sterilization, the test tubes were cooled on a horizontal bench until the media solidified and was ready for use. Each test tube contained one seed, and all operations should be conducted using sterilized forceps on a sterile workbench.

An optimal batch comprising 500 *P. massoniana* seeds was selected for germination. The germinated seeds were placed in a climate-controlled incubator for cultivation. The cultivation temperature was set at 24 °C, and the photoperiod was 12 h of light per day. At 30 d post-germination, pine seedlings were inoculated with LS88.

### 2.3. Experimental Design

Based on the nutrient requirements of pine seedlings and the strain LS88 [23], the inoculation media was prepared by supplementing the germination media. The complete formulation of inoculation media per liter is as follows: 5.8428 mM of sucrose (C_12_H_22_O_11_), 12.8959 mM of potassium phosphate monobasic (H_2_KO_4_P), 16.7903 mM of potassium nitrate (KNO_3_), 991.1696 µM of calcium chloride (CaCl_2_), 25 nM of boric acid (BH_3_O_3_), 13.3323 µM of zinc sulfate (O_4_SZn), 39.1579 nM of copper sulfate (CuO_4_S), 25 µM of ferrous sulfate heptahydrate (FeH_14_O_11_S), 6.6605 µM of glycine (C_2_H_5_NO_2_), 607.8584 nM of pyridoxine hydrochloride (C_8_H_11_NO_3_·HCl), 27.7356 µM of EDTA disodium salt (C_10_H_14_N_2_Na_2_O_8_), 5.1537 mM of ammonium nitrate (NH_4_NO_3_), 9.0762 mM of magnesium sulfate (MgO_4_S), 1.25 µM of potassium iodide (IK), 36.9205 µM of manganese sulphate (MnO_4_S), 303.5159 nM of sodium molybdate (MoNa_2_O_4_), 48.1362 nM of cobalt chloride (Cl_2_Co), 138.7655 µM of inositol (C_6_H_12_O_6_), 74.1246 nM of thiamine hydrochloride (C_12_H_18_C_l2_N_4_OS), 1.0236 µM of niacinamide (C_6_H_6_N_2_O), 5.4304 mM of ammonium tartrate (C_4_H_12_N_2_O_6_), 20.4132 nM of ferric citrate (C_6_H_5_FeO_7_),and 5.2 g of phytagel.

Potato dextrose agar (PDA) is one of the most commonly used fungal culture media. In this experiment, glucose and potato extract were added to the inoculation media to provide the nutrients required for the growth of the LS88. For the high-nutrient media (labeled H), 5 g of potato extract (Beijing Hongrun Baoshun Technology Co., Ltd. (Beijing, China)) per liter and 2.2202 mM of D (+)-Glucose (C_6_H_12_O_6_) were added. For the low-nutrient medium (labeled L), 2.5 g of potato extract per liter and 1.1101 mM of D (+)-Glucose (C_6_H_12_O_6_) were added. The media was aliquoted into culture bottles with dimensions of 130 mm in height, 95 mm in top diameter, 73 mm in bottom diameter, and a volume of 670 mL. One hundred milliliters of the media solution were added to each bottle, which was subsequently sterilized in an autoclave at 121 °C for 30 min. After sterilization, the bottles were placed on an ultraclean bench to cool naturally, allowing the media to solidify for later use.

**Figure 1:**
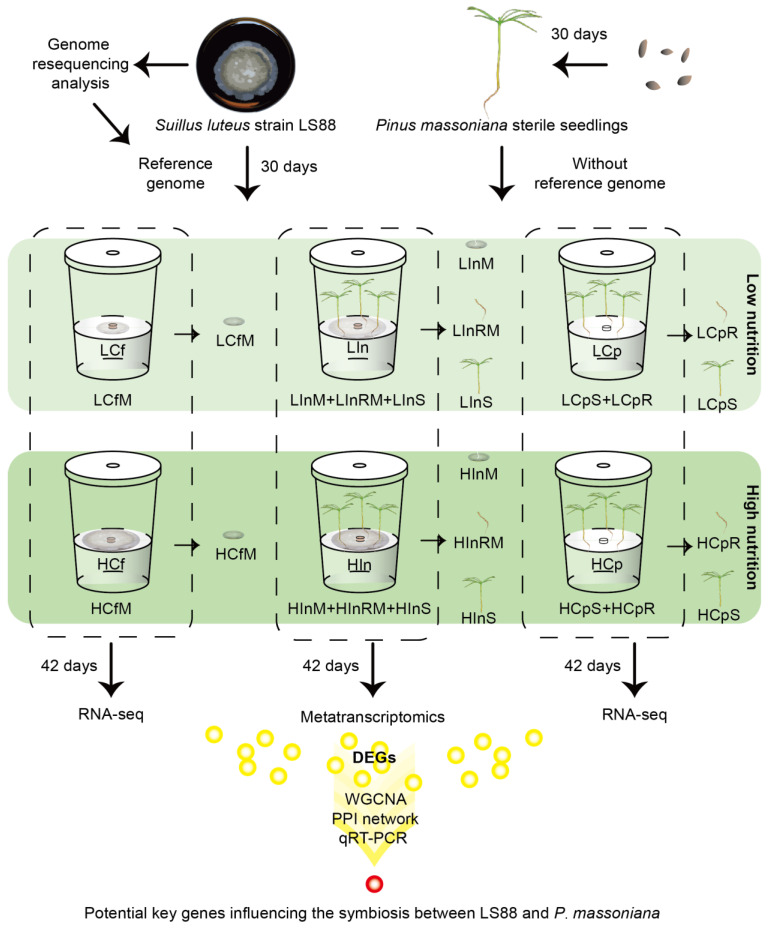
Experimental design.

At 30 d post-germination, uniform and sterile seedlings were transplanted onto the inoculation media surface using forceps, with three seedlings per bottle. The control plant group received a media plug, the inoculation group received an LS88 mycelial plug cultured for 30 d, and the control fungal group contained only an LS88 mycelial plug without any seedlings. The culture bottles were put in a chamber at 24 °C following a 12-h day-night cycle. There were six treatment groups: (1) a high-nutrient control plant group, (2) a high-nutrient control fungal group, (3) a high-nutrient inoculation group, (4) a low-nutrient control plant group, (5) a low-nutrient control fungal group, and (6) a low-nutrient inoculation group. Each treatment group contained 12 culture bottles. Detailed information about each sample can be found in Table 1 and Figure 1.

After being cultured in a climate-controlled incubator for six weeks, the seedlings and fungi in the culture bottles were photographed, the number of survival seedlings was recorded, and samples were harvested. To analyze the effect of LS88 inoculation on different parts of *P. massoniana* seedlings, in the inoculation group, the LS88 mycelia and the roots in contact with mycelia were stored separately from the shoots of *P. massoniana*. Mixed sampling was employed each sample set consisted of three biological replicates. Each replicate included tissue derived from three *P. massoniana* seedlings. The samples were classified, labeled, and frozen in liquid N_2_, followed by storage at −80 °C for subsequent transcriptome analysis. For sample group abbreviations, use M for mycelium, R for roots, S for shoots, Cp for control plant, Cf for fungal, In for inoculation, H for high nutrients, and L for low nutrients, resulting in 12 sample groups. The mycelium used for inoculation and control fungal groups was sampled before treatment. Detailed information about each sample can be found in Table 1 and Figure 1. Furthermore, a portion of the seedling roots was stored in a 50% ethanol solution for future microscopic observation.

Due to the abundant production of dark pigments by LS88 and the difficulty in cross-sectioning delicate sterile seedling roots, trypan blue staining was employed to identify fungal/plant cells and determine the colonization of LS88 in pine roots [25]. The stained samples were then observed using light microscopy with a Zeiss AX10 microscope.

### 2.4. Genome Resequencing Analysis and Annotation of the LS88 Strain

To determine the reference genome for transcriptome sequencing, we resequenced the genome of the LS88 strain and compared it with the existing *S. luteus* whole genome (strain UH-Slu-Lm8-n1, genome ID: 18219) [22]. The DNA from the sample SLG was extracted using the SDS method [26]. The NEBNext^®^ Ultra™ DNA Library Prep Kit for Illumina was used to generate the library. The library was sequenced using the Illumina NovaSeq 6000 platform at Beijing Nuohe Zhiyuan Biotechnology Co., Ltd., Beijing, China (https://cn.novogene.com/, accessed on 6 December 2023). Then, raw reads were trimmed and filtered by FastQC (http://www.bioinformatics.babraham.ac.uk/projects/fastqc/, accessed on 6 December 2023). Then reads were mapped to the reference sequence using BWA (v0.7.8) [27], which was obtained from http://bio-bwa.sourceforge.net/ (accessed on 6 December 2023). To calculate the coverage of the reference sequence and annotate the alignment results, SAMtools (version 0.1.18) [28] was used.

The LS88 genome assembly process involved several steps. First, SOAPdenovo was used for preliminary assembly [29,30], and the resulting sequences were integrated with CISA (http://sb.nhri.org.tw/CISA/en/CISA, accessed on 6 December 2023) [31]. GapCloser (version 1.12) was then employed to modify the assembly results. Finally, the genome of LS88 was assembled at the scaffold level. The genomic information for LS88 has been deposited in the SRA database with ID SRR26883135, and the LS88 genome was used as a reference genome for the next fungal transcriptome analysis. Pipelines for LS88 genome resequencing analysis are shown in Figure S1.

### 2.5. Transcriptome Sequencing and Analysis

Total RNA was extracted from the samples using TRIzol^®^ Reagent, as shown in Table 1 and Figure 1. Every sample group had three biological replicates. The library was sequenced using the Illumina HiSeq 4000 platform at Shanghai Majorbio Co., Ltd., Shanghai, China (http://www.majorbiogroup.com/course, accessed on 6 December 2023). The raw data were processed using SeqPrep (https://github.com/jstjohn/SeqPrep, accessed on 6 December 2023) and Sickle (https://github.com/najoshi/sickle, accessed on 6 December 2023) to obtain clean data. Sequence alignment analysis was performed using TopHat2 [32] and HISAT2 [33].

For transcriptome analysis of the fungal control group (HCf and LCf), the LS88 genome obtained in this study was used as the reference genome, assembled by StringTie version 2.1.2 (https://ccb.jhu.edu/software/stringtie/, accessed on 6 December 2023). Considering that whole genome information for *P. massoniana* was not available, clean data from the plant control groups (HCp and LCp) were used for de novo assembly via Trinity version v2.8.5 [34]. The assembly results were filtered using TransRate version v1.0.3 [35] and Cd-hit version v4.5.7 [36], followed by assembly evaluation using BUSCO version 3.0.2 [37]. Metatranscriptomics was used to sequence the transcriptomes of the samples from the inoculation groups (HIn and LIn). The data were first mapped to the LS88 genome, and then the remaining transcriptomic data were subjected to de novo assembly. The transcriptomic information of this study was submitted to the SRA database under BioProject ID PRJNA1041072. Pipelines for transcriptome sequencing and analysis are shown in Figure S2.

To annotate transcript functions and pathways, various resources and databases were used: Gene Ontology (GO) [38]; Kyoto Encyclopedia of Genes and Genomes (KEGGs) [39,40]; Clusters of Orthologous Groups for Eukaryotic Complete Genomes (KOGs) [41,42]; Non-Redundant Protein Database (NR) [43]; Transporter Classification Database (TCDB) [44]; Pfam [45]; Swiss-Prot [46]; and the Carbohydrate-Active Enzymes Database (CAZy) [47].

Weighted gene co-expression network analysis (WGCNA) can be employed to identify key genes based on their associations with sample groups and gene modules. Genes were grouped into different modules using WGCNA [48,49]. Before the WGCNA of LS88, based on clustering output, we excluded *P. massoniana* shoot samples (HInS and LInS) as well as outlier samples (HInRM1 and LInRM2) from the interaction samples. A soft power (β) of 12 was determined based on the scale independence of the samples. A clustering distance of 0.25 was used. Before the WGCNA of *P. massoniana*, fungal samples (HInM and LInM) and outlier samples (LInRM3, HCpR2, LCpS3, LInS1, HInS2, and HCPS2) were removed based on clustering output. The gene-gene correlation strengths of the remaining samples followed scale independence with a soft power (β) of 6. The clustering distance was set to 0.07.

The gene modules identified by WGCNA were annotated by Gene Ontology (GO) [38] and the Kyoto Encyclopedia of Genes and Genomes (KEGGs) [39,40]. Due to the lack of genome information on *P. massoniana*, the protein-protein interaction (PPI) network analysis was only performed on *S. luteus* through the Search Tool for the Retrieval of Interacting Genes (http://string-db.org/, accessed on 6 December 2023) and visualized using Cytoscape software (version 3.5.1) [50,51].

### 2.6. Other Statistical Analysis

RSEM version 1.3.1 [52] was used for expression quantification to obtain normalized gene or transcript expression levels. The expression levels were quantified using TPM (transcripts per million reads), enabling comparisons of gene expression levels between different samples. Based on the TPM results, a principal component analysis (PCA) can be performed on the samples. The Pearson correlation coefficient (R) is used to analyze the correlation of TPM expression. Based on the corresponding results, we generate the heatmap tree using the complete linkage clustering method with Euclidean distance. The software DESeq2 version 1.24.0 [53] was chosen for the differential expression analysis of the samples. Genes were considered significantly differentially expressed when the fold change exceeded 2 in either direction and the adjusted *p* value was less than 0.05.

To analyze the survival rate of the seedlings, Excel (Office 2019) was used for data preprocessing, and Prism 9.0 was used for generating statistical graphs and conducting one-way ANOVA and two-way ANOVA. The Tukey method was utilized for performing multiple *t*-tests on the data, and the significance levels were <0.05.

### 2.7. Quantitative Reverse Transcription-PCR

To validate the expression of the potential key genes selected by filtered nutrition levels, Tb Green^®^ Premix Ex Taq™ II (Takara, Beijing, China) was used for fluorescence quantitative reverse transcription-PCR (qRT-PCR) analysis, and the hypothetical protein CNBM1630 gene TDF1 was used as the internal reference gene [54]. The primers used for verification of the genes and TDF1 are shown in Table S6. The relative expression of the verified genes was calculated using the 2^−ΔΔCT^ method [55]. All of the data were obtained from three biological replicates, and every biological replicate was tested for three technical replicates.

## 3. Results

### 3.1. Influence of LS88 Inoculation on P. massoniana Seedlings

**Figure 2:**
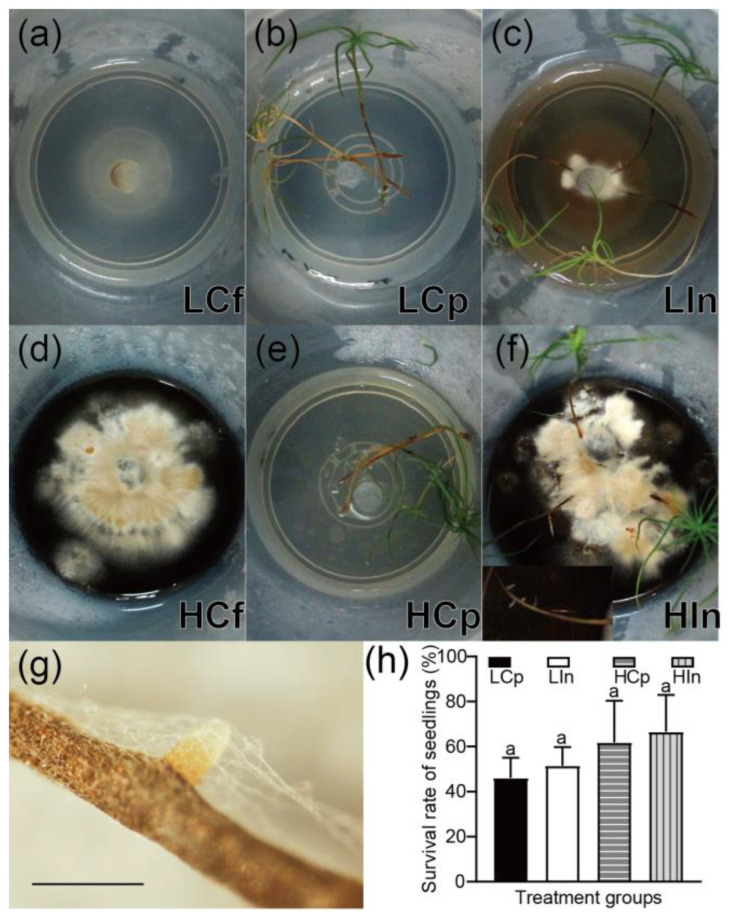
Photos of harvested samples and the survival rate of the seedlings. Note: (**a**) Photo of the low-nutrition fungal control group (LCf); (**b**) Photo of the low-nutrition plant control group (LCp); (**c**) Photo of the low-nutrition inoculation group (LIn); (**d**) Photo of the high-nutrition fungal control group (HM); (**e**) Photo of the high-nutrition plant control group (HCf); (**f**) Photo of the high-nutrition inoculation group (HIn); (**g**) Photos of roots in the high-nutrition inoculation group, bar = 1 mm; (**h**) Survival rate of the seedlings. The data are presented as the means and SEs; n = 36. Values followed by different letters in the same column are significantly different among the treatments at the 0.05 level (determined by an LSD *t*-test).

Under the same nutritional conditions, the inoculation treatment resulted in improved growth of both LS88 and pine seedlings, even in the absence of mature ectomycorrhizae. The growth of the LS88 strain was slow in the low-nutrient medium. In the LCf group (Figure 2a), mycelial growth was sparse, with few mycelia and no aerial mycelia present. In the LCp group (Figure 2b), some seedlings were yellow, and there were no significant changes in the roots of the seedlings compared to those in their initial state (Figure S3d). In the LIn group (Figure 2c), compared to the LCf group, the LS88 strain exhibited better growth. The mycelia were denser, and the formation of dark pigments was observed in the media. The white aerial mycelia appeared on the colony’s surface. In comparison with those in the LCf group, the LIn group exhibited greater seedling survival rates and longer roots, although no fine roots were observed.

The colonies in the HCf group grew well (Figure 2d), fully covering the surface of the culture medium after six weeks. Abundant aerial mycelia were visible (Figure 2d). In the HCp treatment group, the growth of *P. massoniana* seedlings (Figure 2e) was greater than that in the LCp treatment group (Figure 2b). The roots of the seedlings in the HIn group were more developed than those in the other groups, with longer primary roots and numerous fine roots developing, many of which were enveloped by the mycelia of strain LS88. Observation under a dissection microscope revealed a mycelial net covering the fine roots of the HIn group (Figure 2g).

In the high-nutrient culture medium (Figure 2h), LS88 inoculation increased the survival rate by 4.77%. Under low-nutrient conditions, LS88 inoculation increased the survival rate by 5.37%. The results of the statistical tests indicate that there is no significant difference between the groups. Figure S1 displays stained images of inoculated roots, revealing the association between LS88 and pine roots. However, mature ECM structures were not observed.

### 3.2. Genome and Transcriptome Sequencing Statistics

The size of the *S. luteus* strain LS88 genome is 43.17 Mb, has a GC content of 47.83% in scaffold, and it includes 134 tRNAs. The SRA ID of the LS88 genome is SRR26883135.

According to the quality control results, the samples from the LCf group had insufficient RNA for transcriptomic analysis, possibly due to poor fungal growth within this group (Figure 2a). Therefore, these samples (LCfM1-3) were not used for subsequent transcriptome analysis. A summary of the other transcriptome sequencing data is shown in Tables S1 and S2. Table S3 displays the functional annotation statistics for the expressed transcripts. As shown in the tables, after filtering the raw reads, the error rate of the clean reads was less than 0.03%. The Q20 value for the clean reads was above 95%, and the Q30 value was above 90%. The sequencing quality of the samples met the requirements for subsequent analysis. The corresponding SRA numbers are listed in Tables S4 and S5.

### 3.3. Analysis of Sample Transcriptome Expression Levels

**Figure 3:**
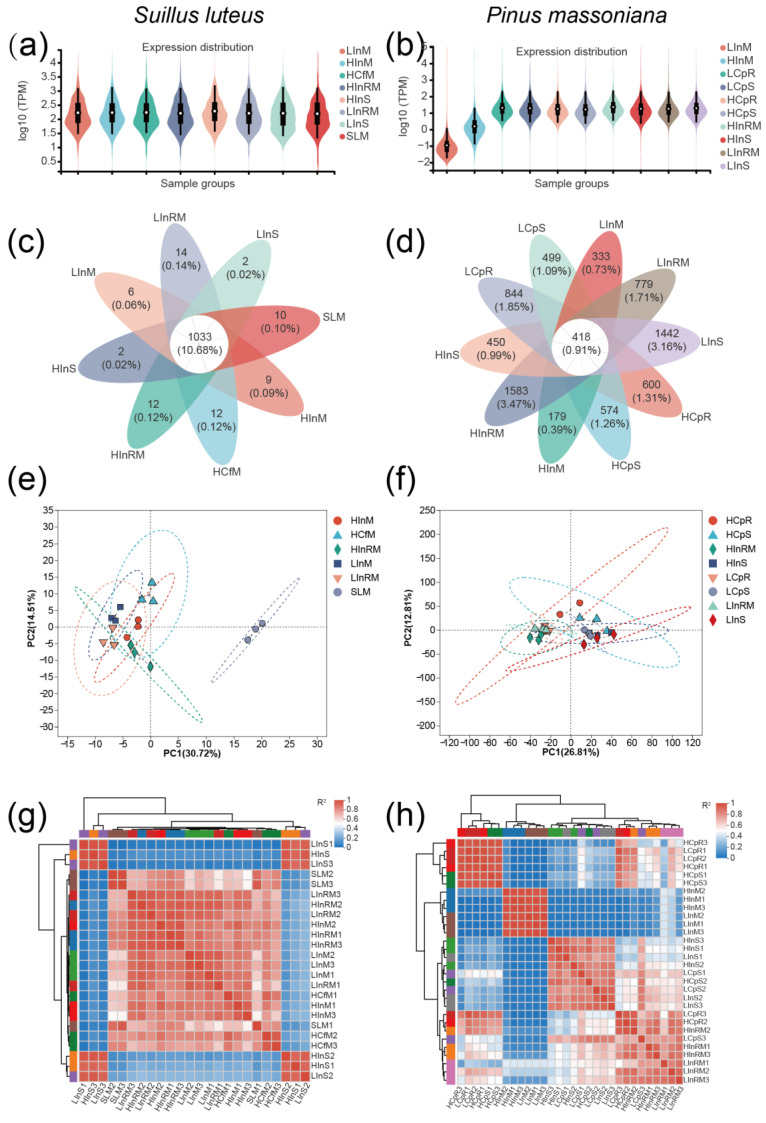
Gene expression analysis of the samples. Note: (**a**) Violin plot of expression distribution based on fungal transcriptome; (**b**) Violin plot of expression distribution based on pine transcriptome; (**c**) Venn diagram analysis of expression between groups based on fungal transcriptome; (**d**) Venn diagram analysis of expression between groups based on pine transcriptome; (**e**) PCA between sample groups based on fungal transcriptome; (**f**) PCA between sample groups based on pine transcriptome; (**g**) correlation heatmap between samples based on fungal transcriptome; (**h**) correlation heatmap between samples based on pine transcriptome.

The LS88 transcriptome expression of samples is primarily influenced by inoculation and tissue types, with a lower impact from nutrient levels. In terms of pine transcriptome expression, the effect of LS88 inoculation on pine trees appears to be greater under high-nutrient conditions. As shown in Figure 3a, the transcriptome expression levels of LS88, including those of shoot samples (HInS and LInS), in the inoculation group exhibited similar patterns. According to the *P. massoniana* transcriptome expression distribution (Figure 3b), pine transcriptomic information found in the mycelium is not associated with the root (HInM and LInM), with the lowest expression level observed in the LInM group. This suggests that LS88 and *P. massoniana* contain numerous homologous genes. Increasing nutrient levels helps the expression of pine transcripts in LS88 upon contact, even without the establishment of ectomycorrhiza.

As Figure 3c shows, the LS88 transcriptome exhibited a greater number of shared genes across different sample groups, with a total of 1033 sequences. In contrast, the *P. massoniana* transcriptome included more unique genes, the HInRM group had the greatest number of unique genes (Figure 3d). In comparison to the LInRM samples, the HInRM samples showed higher pine transcriptomic expression and a lower number of active unique genes. This finding suggests a deeper level of interaction between LS88 and pine roots under high-nutrient conditions, indicating a potential stabilization phase where gene expression has transitioned beyond the stage of dramatic changes.

Due to the fact that the HInS and LInS samples were positioned far from the other samples in the PCA analysis based on the LS88 transcriptome, the LS88 transcriptome PCA plot displayed in the figure represents the results of the other samples (Figure 3e). As a result, the pine transcriptome PCA plot displayed in the figure represents the results besides the LInM and HInM samples. According to the analysis of the fungal transcriptome, the impact of inoculation on the clustering of samples outweighs the influence of nutrient levels. The fact that the LInM samples are closer to the HCfM samples than the HInM samples suggests that LS88 and pine may have a deeper level of interaction in the HIn treatment group. Furthermore, according to the pine transcriptome analysis (Figure 3f), Additionally, according to the analysis of the pine transcriptome, the samples are separated based on the aboveground and belowground parts of the seedlings. Interestingly, it seems that increasing nutrient conditions exacerbates the impact of inoculation on the samples compared to low-nutrient conditions.

Based on the fungal transcriptome correlation heatmap (Figure 3g), two shoot groups have low correlations with other groups, while strong correlation exists among biological replicate samples in the remaining groups. In the pine transcriptome correlation heatmap (Figure 3h), samples are differentiated based on tissue type, nutrient levels in the media, and whether they were inoculated.

### 3.4. Analysis of Differential Gene Expression

The analysis of the number of differentially expressed genes (DEGs) based on the LS88 and pine transcriptomes, when the significance levels (*p* adjusted) were <0.05, indicates a stronger effect of the inoculation treatment on transcriptional expression in the samples compared to nutrient changes in root and mycelium samples. To validate our hypothesis, we aim to rapidly screen potential key genes associated with ECM development between LS88 and *P. massoniana* by creating nutrient differences. In further analysis, we expect to identify genes that exhibit consistent trends in expression changes between control and inoculated groups under varying nutrient levels.

**Figure 4:**
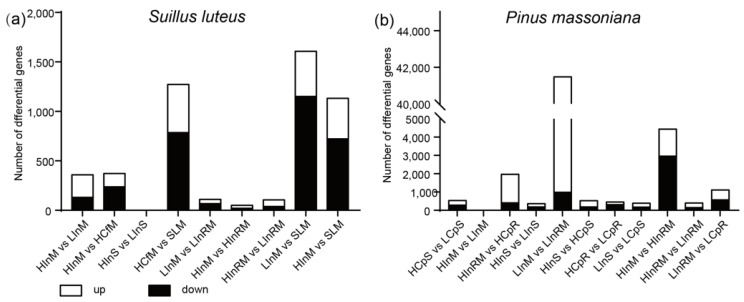
Statistics of differential gene expression between different comparison groups with significance levels (*p* adjusted) were <0.05 based on the *S. luteus* (**a**) and *P. massoniana* (**b**) transcriptomes.

As shown in Figure 4a, there were no DEGs between HInS and LInS, indicating that nutrient levels did not significantly influence LS88 transcriptome expression in the inoculated pine shoots in this study. On the other hand, the number of DEGs between HInM and LInM and between HInM and HCfM is similar. This suggests that in this experiment, reducing nutrient levels increases the number of upregulated genes in the mycelium, while inoculation under high-nutrient conditions leads to an increase in the number of downregulated genes in the mycelium. The number of DEGs between the HInM and HInRM groups was lower than that between the LInM and LInRM groups, implying that when the duration of the inoculation treatment is consistent, the reduction in nutrient levels results in an amplified difference in transcriptome expression between the mycelium associated with pine roots and the mycelium not associated with pine roots.

Based on the pine transcriptome (Figure 4b), there were no DEGs between the HInM and LInM groups, and the significance levels (*p* adjusted) were <0.05, indicating that the nutrient differences set in this study did not significantly affect the pine transcriptome expression in the mycelium sample. Conversely, the differential gene expression between HInM and HInRM was much lower than that between LInM and LInRM, suggesting that under low-nutrient conditions, the LS88 strain induces more substantial changes in signaling pathways within pine roots, which is correlated with an increase in DEGs. In the HInM and HInRM comparison groups, downregulated genes were predominant (66.62% of DEGs), indicating increased signal communication between LS88 and *P. massoniana* roots under high-nutrient conditions, potentially leading to the incorporation of numerous transcripts from *P. massoniana* roots into the LS88 mycelium. Additionally, HCPS and LCpS exhibited more DEGs than HInS and LInS did, suggesting that nutrient levels may have a greater impact on *P. massoniana* shoots, whereas the influence of the LS88 strain may outweigh nutrient effects in *P. massoniana* roots.

Due to the lack of genome information on *P. massoniana*, this study shifted its focus to further transcriptome analysis of *S. luteus*. Additional comparative analysis (Table 2) confirmed that in the high- and low-nutrition inoculation root and mycelium samples (HIn and LIn treatment groups), four genes were significantly upregulated in the LInM vs. LInRM and HInM vs. HInRM comparisons. This finding implies that the variations in these genes are influenced mainly by the host and that they are potential key genes involved in the mycorrhizal association mechanism. Among them, only one gene, evm.TU.Scaffold330.4 (ScaffoldD), was significantly upregulated in the HCfM vs. HInM comparison, suggesting that the expression of this gene in *S. luteus* is inducible by *P. massoniana*. Based on the annotation results from the KEGG, Swiss-Prot, and Pfam databases, the putative function of ScaffoldD is the encoding of a glutathione S-transferase (GST). According to the annotation in the NR database, the annotation result for ScaffoldD is a hypothetical protein CY34DRAFT_811040 (KIK36730.1) from the *S. luteus* strain UH-Slu-Lm8-n1 [22].

There were two genes, namely, evm.TU.Scaffold679.5 (ScaffoldE) and evm.TU.Scaffold806.1 (ScaffoldF), which exhibited significant decreases in expression in the LInM vs. LInRM and HInM vs. HInRM comparisons. However, these genes did not significantly change according to the other comparisons. Interestingly, ScaffoldE was not detected in the shoots of *P. massoniana*. The database used in this study does not provide a putative function for ScaffoldE. According to the annotation results from the GO database, ScaffoldF may be associated with histone acetylation, histone acetyltransferase complex, and kinase activity. However, according to the KEGG annotation results, the gene is annotated as a transformation/transcription domain-associated protein (K08874, TRRAP). In the Swiss-Prot database, it can be annotated as sp|P38811|TRA1_YEAST. According to the annotation in the NR database, the annotation result for ScaffoldF is a hypothetical protein CY34DRAFT_797712 (KIK48922.1) from the *S. luteus* strain UH-Slu-Lm8-n1.

### 3.5. Transcriptome-Weighted Gene Co-Expression Network Analysis

Through WGCNA, potential key genes related to the ECM interaction between LS88 and *P. massoniana* were identified, and the possible functions of the previously screened LS88 genes were evaluated. In this study, by analyzing the correlations between gene modules and performing an integrated plant phenotype analysis, we inferred that certain modules are associated with *P. massoniana* root development, photosynthesis, and interactions with the LS88 strain. Moreover, based on the comparison of significantly upregulated genes between the interaction samples and fungal samples, we identified modules that are potentially associated with LS88 recognition and colonization of *P. massoniana*.

**Figure 5:**
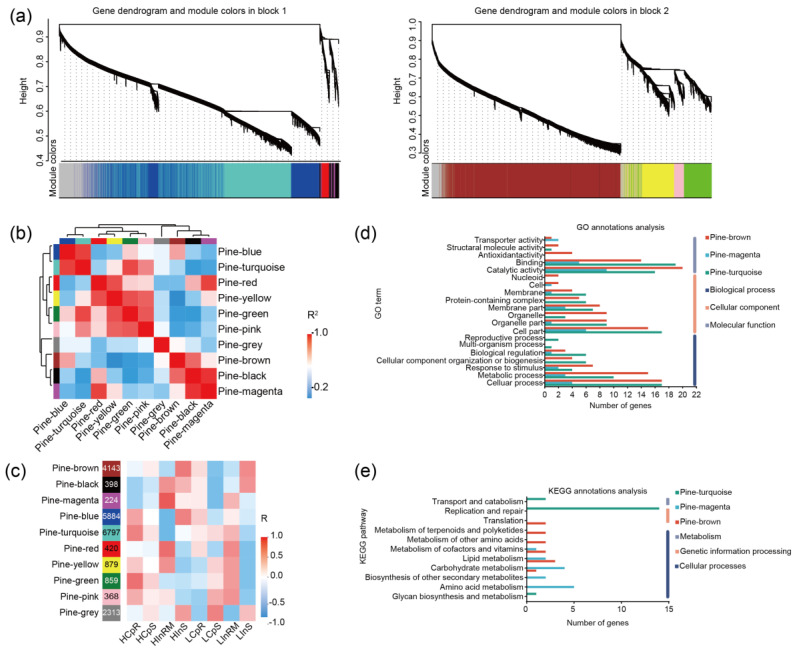
WGCNA based on the *P. massoniana* transcriptome. Note: (**a**) Correlations among modules; (**b**) Correlations between modules and traits; (**c**) Gene dendrograms and module colours; (**d**) GO terms distribution of the top 30 genes in connectivity in network analysis based on the fungal-magenta, pine-turquoise, and pine-brown modules; (**e**) KEGG annotations of the top 30 genes in connectivity in network analysis based on the pine-magenta, pine-turquoise, and pine-brown modules.

Figure 5 presents the results of the WGCNA analysis of the *P. massoniana* transcriptome. The genes were divided into ten modules. Figure 5a illustrates the module classification tree, which has been split into two parts due to the large number of genes involved (22,285). Figure 5b illustrates the inter-module correlation of the gene modules. Pine-turquoise and pine-blue displayed strong correlations, while pine-grey showed a weaker correlation with the other modules. According to the clustering analysis, pine-grey, pine-brown, pine-black, and pine-magenta clustered together, while the other six gene modules formed a separate branch. Figure 5c shows the correlation between the gene modules and the *P. massoniana* sample group. Pine-grey was positively correlated with the *P. massoniana* shoot samples. The pine-green module was negatively correlated with HInRM and LInRM, exhibiting an opposite relationship with the other root samples (HInRM and LInRM) from the control group, indicating a potential relationship between this module and the colonization of *P. massoniana* roots by LS88.

The roots from the inoculation group showed a positive correlation with pine-magenta. Pine-turquoise showed a significant positive correlation with HCpR and a significant negative correlation with HInRM, suggesting its relevance to the response of *P. massoniana* during this interaction phase. pine-magenta was positively correlated with the LInRM and was significantly positively correlated with the HInRM (*p* = 0.0038). The HInRM roots exhibited significant developmental improvement with lateral root growth, while the LInRM primary roots elongated but did not form lateral roots. This finding suggested a potential relationship between pine-magenta and root elongation and development. Pine-brown was strongly positively correlated with the *P. massoniana* shoot samples and negatively correlated with the LCpS group. This finding suggested a potential association between pine-brown and photosynthesis in *P. massoniana*.

Based on the network analysis of the pine-magenta, pine-turquoise, and pine-brown modules, the top 30 genes in terms of connectivity were identified, and three gene sets were established. The GO and KEGG databases were used for annotation analysis. GO analysis (Figure 5d) of the magenta, turquoise, and brown gene sets revealed that these genes were enriched in biological processes, cellular components, and molecular functions. In the brown and magenta gene sets, most genes were related to catalytic activity. On the other hand, in the turquoise gene set, the greatest number of genes were related to binding. Based on the KEGG annotation results (Figure 5e), the magenta gene set appears to be predominantly enriched in cellular component functions. The turquoise gene set was enriched in metabolism-related functions, while the brown gene set was enriched in lipid metabolism functions.

**Figure 6:**
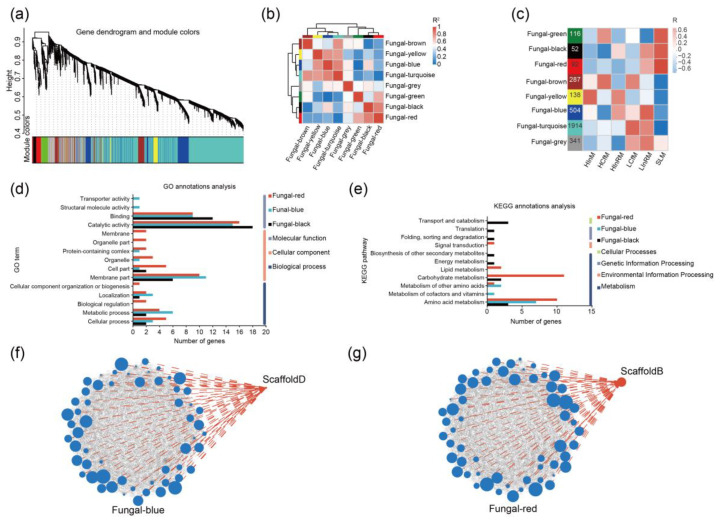
WGCNA based on the *S. luteus* transcriptome. Note: (**a**) Correlations between modules; (**b**) Correlations between modules and traits; (**c**) Gene dendrograms and module colours; (**d**) GO terms distribution of genes in the fungal-red, fungal-blue, and fungal-black modules; (**e**) KEGG annotations of genes in the fungal-red, fungal-blue, and fungal-black modules; (**f**) Network visualization of interactions in the fungal-blue module; (**g**) Network visualization of interactions in the fungal-red module.

Figure 6 shows the WGCNA of the LS88 transcriptome. Figure 6a shows the hierarchical clustering tree of LS88 modules generated by WGCNA. As shown in Figure 6b, genes were clustered into eight modules, each representing distinct expression patterns. Figure 6c shows the correlation between different module genes and sample groups. The fungal-green, fungal-black, and fungal-red modules showed positive correlations with the SLM group, indicating higher expression levels of these genes in the LS88 mycelium grown under liquid culture conditions. In the low-nutrient inoculation group, fungal-blue, fungal-turquoise, and fungal-grey were positively correlated with mycelia growing inside and outside *P. massoniana* roots (LInRM and LInM, respectively). Additionally, fungal-black and fungal-blue were positively correlated with HInRM and LInRM, suggesting that these modules are influenced mainly by the degree of interaction between LS88 and *P. massoniana* roots.

Among the four genes listed in Table 2, ScaffoldB belongs to the fungal-red module, while the remaining genes belong to the fungal-blue module. This finding suggested a potential association between ScaffoldB and the recognition mechanisms of LS88 and *P. massoniana* and a possible correlation between the fungal-blue module and LS88 colonization in host roots. The significantly downregulated gene ScaffoldE belongs to the fungal-black module, while ScaffoldF does not belong to any module.

Based on the network analysis of the fungal-blue, fungal-red, and fungal-black modules, the top 30 genes in terms of connectivity were identified, and three gene sets were established. According to Figure 6d, the gene enrichment analysis in the GO database indicated that there was a greater number of genes enriched in the molecular function category than in the cellular component and biological process categories. The KEGG annotation results indicated that these genes are primarily associated with metabolism (Figure 6e). The KEGG annotation information for ScaffoldD indicates its association with glutathione metabolism, with the KO ID being K00799. The GO annotation information suggested its relevance to transferase activity, with a GO ID of 0016740. The GO annotation information for ScaffoldB suggested its involvement in molecular functions. It was annotated with several GO IDs, including 0005506, 0016491, 0016705, and 0031418. The most likely function is oxidoreductase activity. However, there is no KEGG annotation information available for this gene.

Network analysis of the fungal-blue module (Figure 6f) revealed that the connectivity of ScaffoldD was 113.833, which was 57th among the genes analyzed. The fungal-blue module consisted of a total of 504 genes, with the highest connectivity value being 146.392. The fungal-red module comprised 92 genes (Figure 6g), with the highest connectivity value being 24.011. The gene ScaffoldB has a connectivity value of 14.428 and is ranked 25th among the genes in the module.

### 3.6. Protein-Protein Interaction Analysis and qRT-PCR Results of Potential Key Genes

**Figure 7:**
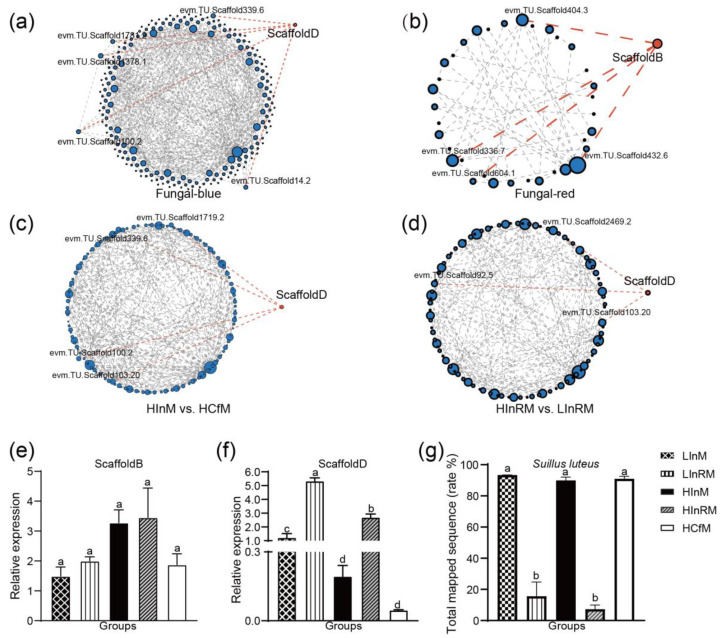
PPI analysis and qRT-PCR results of potential key genes. Note: (**a**) PPI analysis based on genes from the fungal-blue module; (**b**) PPI analysis based on genes from the fungal-red module; (**c**) PPI analysis based on DEGs from the HCfM vs. HInM comparisons; (**d**) PPI analysis based on DEGs from the HInRM vs. LInRM comparisons; (**e**) qRT-PCR results of ScaffoldB in the samples, the data are presented as the means and SEs; n = 9; (**f**) qRT-PCR results of ScaffoldD in the samples, the data are presented as the means and SEs; n = 9; (**g**) sequence mapped rate to LS88 genome in samples, the data are presented as the means and SD; n = 3. Values followed by different letters in the same column are significantly different among the treatments at the 0.05 level (determined by an LSD *t*-test).

To validate the function of the previously identified potential key genes, Protein–protein interaction (PPI) analysis was employed to analyze the specific gene sets of *S. luteus*. In the fungal-blue module (Figure 7a) gene set, all genes had a combined score greater than 0.4. However, in the fungal-red module gene set, 45 genes had a combined score greater than 0.4, while only 11 genes in the fungal-red module (Figure 7b) had a combined score greater than 0.4. Based on the PPI analysis of the fungal-red module gene set, only the gene ScaffoldD appeared in the results. Moreover, ScaffoldD was linked to five genes, with the highest combined score of 0.776 observed with evm.TU.Scaffold1378.1; both genes were annotated to the KEGG pathway map00480. In the fungal-red module, ScaffoldB was connected to four genes, with the highest combined score (0.485) observed with evm.TU.Scaffold336.7. This gene could be annotated with both GO terms: biological process and molecular function.

In the PPI analysis, which was based on comparisons of DEGs between HCfM vs. HInM as well as HInRM vs. LInRM, the results revealed only the presence of ScaffoldD among the potential genes mentioned. This indicates that ScaffoldD is a core gene within these two gene sets. The relative expression levels of the genes ScaffoldD and ScaffoldB were determined via qRT-PCR, and the expression trends of both genes in the samples agreed with the transcriptomic results (Table 2 and Figure 7e,f). Notably, the expression of ScaffoldD in LInRM was significantly higher than in HInRM, while the read mapped rate of the LS88 genome in LInRM was higher than in HInRM. This suggests that the expression of this gene is probably induced by *P. massoniana*. The two-way ANOVA analysis results of relative expression levels of ScaffoldD and sequence-mapped rate to the LS88 genome in LInRM and HInRM showed a *p*-value of interaction of 0.062. According to the alignment results on NCBI, the comparison of gene and transcript sequences for ScaffoldD is presented in Tables S7 and S8.

## 4. Discussion

### 4.1. LS88 Enhances P. massoniana Growth without Mature Ectomycorrhizae

Without mature ECM structures, LS88 still enhances *P. massoniana* growth. Inoculation with LS88 improved the survival rate and growth status of the *P. massoniana* seedlings (Figure 2). *P. massoniana* developed fine roots in the inoculation treatment (Figure 2f). Previous studies have shown that inoculation with *S. luteus* can increase the total root length, surface area, root tip number, and root volume of *P. massoniana* seedlings [18].

Similar Hartig net-like structures enclosing root hair cells were also observed in the mature zone of *P. massoniana* (Figure S4). However, mature *S. luteus–P. massoniana* ECM structures were not observed. Nevertheless, in a pot experiment, the LS88 strain was able to form mature ECM structures with *P. massoniana* seedlings [23]. This discrepancy may be attributed to limitations in the cultural environment. Similar phenomena have been reported in studies related to *Paxillus involutus*; in the absence of mature ECM structures, these fungal strains can still promote host growth and assist in stress tolerance [56,57,58,59,60]. This study confirmed the significant growth-promoting effect of the LS88 strain on *P. massoniana*, providing evidence for potential signaling exchange between the two species during interaction.

### 4.2. The Role of Nutrients in the LS88 Colonization of P. massoniana

The addition of organic substances can influence the development of plants and AMF [61]. Similar observations were made in this study: an increase in nutrient levels in the culture media enhanced the positive effects of LS88 on *P. massoniana*, resulting in a more developed root system (Figure 2f). In contrast, the high nutrient control plants did not exhibit any observable fine roots (Figure 2e). Our previous research also demonstrated that adding organic fertilizers enhanced the growth-promoting effects of LS88 on *P. massoniana* [23]. Similar findings have been reported for arbuscular mycorrhizal and endophytic fungi [62,63,64].

Reducing the nutrient content of the culture medium facilitated the colonization of *P. massoniana* roots by LS88 (Table S1), and the gene expression of the mycelium is more active (Figure 4a). Our results indicate that the growth of the seedlings inoculated with LS88 improved compared to that of those not inoculated with LS88 in low nutrient media (Figure 2b,c). The different parts of *P. massoniana* responded differently to nutrients and LS88. In the case of *P. massoniana* shoots, altering nutrient levels resulted in greater differences in gene expression than did LS88 inoculation. Conversely, for *P. massoniana* roots, high-nutrient cultivation conditions enhanced the responsiveness of the plants to LS88 after six weeks of inoculation (Figure 4b). Similar phenomena have been reported in studies on *Rhizopogon occidentalis* and *Pinus muricata*. It has been found that *R. occidentalis* tends to preferentially colonize the roots of *P. muricata* in nutrient-rich environments [65]. In our pot experiment, only the inoculation group supplemented with organic fertilizer yielded mature mycorrhizae [23]. Considering that both *R. occidentalis* and LS88 belong to the Suillineae family, it can be hypothesized that LS88 employs a similar colonization strategy as *R. occidentalis* by establishing itself in the roots during the early growth stage and adapting better to high-nutrient conditions.

Considering the nutrient response of LS88 and *P. massoniana* in the subsequent artificial cultivation of *S. luteus*, a two-step approach can be adopted. Initially, LS88 can be inoculated into the roots of *P. massoniana* under low-nutrient conditions to establish a symbiotic relationship. After an initial period of inoculation, nutrient levels can be increased to promote the development and maturation of ectomycorrhizae.

### 4.3. Potential Key Genes Influencing the Symbiosis between LS88 and P. massoniana

By manipulating nutrient levels to create differences, we performed a rapid screening of significant differentially expressed genes (DEGs) in the sample groups and identified a potential candidate gene in *S. luteus* that can be used as a marker to determine the colonization of LS88 in pine tree roots., namely, ScaffoldD. This gene likely encodes a glutathione S-transferase (GST), as suggested by Lofgren et al. [66]. Previous studies have demonstrated the crucial role of GSTs in the interaction between plants and fungi [67]. Proteins from this family can also alleviate metal stress in endophytic fungi [68]. Similar analyses have been recently applied in the study of interactions between mycorrhizal fungi and their hosts [69,70,71].

In recent years, WGCNA has been widely utilized for analyzing the response of mycorrhizal fungi to the environment and their host [72,73]. Although the protein sequence of ScaffoldD can be annotated to the hypothetical protein sequence of *S. luteus* UH-Slu-Lm8-n1 in the NR database, with the accession number KIK36730.1 [22], there have been no prior reports of similar coding sequences in *S. luteus*. However, records of similar coding sequences have been found in other *Suillus* spp. [66]. In further studies on ECM development between *S. luteus* and *P. massoniana*, we will utilize phenotypic data, transcriptomic analysis, and metabolomic analysis to investigate the impact of exogenous GST. This will involve measuring GST activity in the fungal and pine samples, as well as exploring the genes associated with GST biosynthesis.

As an emerging model genus, extensive data on the *Suillus* fungi has been accumulated in recent years [11]. As the type species of the *Suillus* genus, research has been conducted on *S. luteus* regarding its role in promoting host plant growth and metal tolerance [23,74,75,76]. The results of our study provide new insights for the rapid identification of key genes involved in the ectomycorrhizal association between *S. luteus* and its host. A potential marker for *S. luteus* colonization, the GST gene, can potentially be used to assess the degree of *S. luteus* colonization on the host, and the related methods can be applied in vitro, in pot, and even in field investigations. Even before ectomycorrhizal development matures, this method could be useful for quantifying the extent of *S. luteus* colonization on host roots.

## 5. Conclusions

In conclusion, our study utilized subtle variations in nutrient levels to perform a rapid identification of genes in the *Suillus luteus* strain SL88 that showed expression patterns influenced solely by the inoculation treatment with *Pinus massoniana*. Through this approach, we identified a GST gene that is associated with *S. luteus* colonization. Analysis of the transcriptomic changes in the roots and aerial parts of *P.* seedlings in response to *S. luteus* strain LS88 under high- and low-nutrient conditions revealed that at 42 d post-inoculation, LS88 inoculation did not significantly alter gene expression in the aerial parts of *P. massoniana* seedlings, but it had a greater impact on gene expression in the roots than did nutrient manipulation. Nutrient limitation promoted the colonization of *P. massoniana* roots by LS88. However, enhancing the growth of LS88 and *P. massoniana* seedlings and the development of mycorrhizae required additional nutrients. Furthermore, through integrated analysis of the genome and transcriptome, we identified one LS88 gene associated with GST, suggesting that glutathione metabolism could be a potential key pathway involved in LS88 colonization of the host. This GST gene (ScaffoldD) has the potential to serve as an indicator of *S. luteus* colonizing *P. massoniana*.

## Figures and Tables

**Table 1:** Sample and treatment group code information.

Media Types	Treatment Groups	Sample Groups	Sample Description
High nutrition	High nutrient control plant group (HCp)	HCpS	Shoots
		HCpR	Roots
	High nutrient control fungal group (HCf)	HCfM	mycelium
	High nutrient inoculation group (HIn)	HInM	mycelium
		HInRM	Roots associated with mycelium
		HInS	Shoots
Low nutrition	Low nutrient control plant group (LCp)	LCpS	Shoots
		LCpR	Roots
	Low nutrient control fungal group (LCf)	LCfM	mycelium
	Low nutrient inoculation group (LIn)	LInM	mycelium
		LInRM	Roots associated with mycelium
		LInS	Shoots
Strain LS88	SL	SLM	Mycelium used for transcriptome analysis
Strain LS88	SL	SLG	Mycelium used for genome analysis

**Table 2:** Fungal genes were significantly upregulated between interaction samples and fungal samples.

Gene Id	LInM vs. LInRM	HInM vs. HInRM	HCfM vs. HInM
evm.TU.Scaffold161.2 (ScaffoldA)	Upregulated *	Upregulated *	Upregulated
evm.TU.Scaffold2140.1 (ScaffoldB)	Upregulated *	Upregulated *	Downregulated
evm.TU.Scaffold1771.2 (ScaffoldC)	Upregulated *	Upregulated *	Upregulated
evm.TU.Scaffold330.4 (ScaffoldD)	Upregulated *	Upregulated *	Upregulated *

Note: * *p* adjusted < 0.05.

## Data Availability

The data, strain LS88, and pipelines used in this study can be made available upon request.

## Supplementary Materials

The following supporting information can be downloaded at: https://www.mdpi.com/article/10.3390/jof10080587/s1, Table S1: Summary of transcriptome sequencing data and alignment between sample clean reads and the LS88 genome; Table S2: Summary of the transcriptome sequencing data and alignment between the sample clean reads and *P. massoniana* assembly transcripts; Table S3: Summary of transcriptomic functional annotations; Table S4: Transcriptomic submission information of LS88; Table S5: Transcriptomic submission information of *P. massoniana*; Table S6: Primers for potential key genes and TDF1; Table S7: Gene sequence alignment results on NCBI; Table S8: Transcript sequence alignment results on NCBI; Figure S1: Pipelines for LS88 genome resequencing analysis title; Figure S2: Pipelines for transcriptome sequencing; Figure S3: Stained pictures of inoculated roots; Figure S4: Cultivation of sterile *P. massoniana* seedlings.
