# Supplementary material for: Differential Strategies of Ectomycorrhizal Development between Suillus luteus and Pinus massoniana in Response to Nutrient Changes
Source: J Fungi (Basel). 2024 Aug 19;10(8):587. doi: 10.3390/jof10080587 (PMC11355094; doi:10.3390/jof10080587)
Supplement: Supplementary file 1 [file jof-10-00587-s001.zip › Supplementary Materials.pdf]

Supplementary Materials

Title: Differential Strategies of Ectomycorrhizal Development between *Suillus luteus* and *Pinus massoniana* in Response to Nutrient Changes

Authors: Xueyu Pan [1,2,3,†], Junfeng Liang[2,†], Jinhua Zhang [2], Yan Zhao [1,*] and Mingjie Chen [1,*]

Affiliations:

[1] Institute of Edible Fungi, Shanghai Academy of Agricultural Sciences, Shanghai 201403, China; panxueyu@saas.sh.cn (X.P.)
[2] Research Institute of Tropical Forestry, Chinese Academy of Forestry, Guangzhou 510520, China; jfliang2000@163.com (J.L.); jhzhang0111@163.com
[3] Shanghai Key Laboratory of Agricultural Genetics and Breeding, Shanghai Academy of Agricultural Sciences, Shanghai 201106, China
* Correspondence: jiandan289@126.com (Y.Z.); mjchen@saas.sh.cn (M.C.)
† These authors contributed equally to this work.

The following Supporting Information is available for this article:

Table S1 Summary of transcriptome sequencing data and alignment between sample clean reads and the LS88 genome

Sample	Raw reads	Clean reads	Error (%)	Q20 (%)	Q30 (%)	GC content (%)	Total mapped (rate %)
HInM1	77867058	77189782	0.0246	98.12	94.54	51.07	70670064(91.55)
HInM2	78657284	78023582	0.0246	98.16	94.54	50.72	68371120(87.63)
HInM3	75756874	75239004	0.0244	98.25	94.76	51.11	68251980(90.71)
HCfM1	43551184	43232918	0.0245	98.23	94.7	51.04	39819673(92.1)
HCfM2	40853564	40312074	0.0245	98.21	94.66	50.97	35937988(89.15)
HCfM3	44415212	43933444	0.0248	98.08	94.37	50.94	40224340(91.56)
HInRM1	75854892	74540946	0.0273	97.12	92.07	45.34	3106625(4.17)
HInRM2	73835726	73215038	0.0247	98.13	94.38	45.96	6584480(8.99)
HInRM3	83519260	82878820	0.0246	98.19	94.51	45.94	7219597(8.71)
HInS1	69689436	69092988	0.0247	98.16	94.45	46.04	7615(0.01)
HInS2	70573962	70029700	0.0248	98.14	94.35	45.71	5677(0.01)
HInS3	70208228	69684122	0.0244	98.25	94.69	46.55	1953(0.00)
LInM1	80776968	80296594	0.0238	98.56	95.24	50.95	74925152(93.31)
LInM2	90347812	89775160	0.0237	98.6	95.39	51.00	83897976(93.45)
LInM3	86855030	86342260	0.0238	98.58	95.34	50.99	80660908(93.42)
LInRM1	85388192	84805734	0.0241	98.47	94.96	46.36	11654691(13.74)
LInRM2	79666866	79096722	0.0241	98.45	94.91	47.01	20179780(25.51)
LInRM3	91861808	91095768	0.0242	98.44	94.88	46.08	6970519(7.65)
LInS1	87494816	86881636	0.0242	98.44	94.87	46.09	704(0.00)
LInS2	89791100	89139106	0.024	98.51	95.02	45.37	11310(0.01)
LInS3	83511642	82953138	0.0242	98.43	94.79	45.56	2117(0.00)
SLM1	40313058	40022150	0.0254	97.88	93.72	51.05	38539013(96.29)
SLM2	43217538	42944058	0.0255	97.85	93.67	51.01	41140992(95.8)
SLM3	41288076	40975300	0.0255	97.83	93.6	51.04	39352441(96.04)

Table S2 Summary of the transcriptome sequencing data and alignment between the sample clean reads and *P. massoniana* assembly transcripts

Sample	Raw reads	Clean reads	Error (%)	Q20 (%)	Q30 (%)	GC content (%)	Mapped reads (rate %)
HCpR1	51678988	25626195	0.0243	98.32	94.85	45.79	20379761 (79.53)
HCpR2	43994962	21768351	0.0249	98.04	94.3	45.46	17012096 (78.15)
HCpR3	42053662	20819660	0.0249	98.09	94.25	45.69	16421225 (78.87)
HCpS1	44771590	22184914	0.0244	98.24	94.7	45.89	17798881 (80.23)
HCpS2	47616178	23627770	0.0243	98.3	94.81	45.73	18951540 (80.21)
HCpS3	51655514	25599971	0.0247	98.17	94.47	46.50	19898234 (77.73)
HInM1	4771886	2385836	0.0294	95.84	90.81	49.39	1182911 (49.58)
HInM2	8040244	4019939	0.0256	97.64	93.67	47.41	2714895 (67.54)
HInM3	5227130	2613450	0.0258	97.56	93.59	49.98	1161952 (44.46)
HInRM1	71251168	35621605	0.0273	97.12	92.05	45.08	27532708 (77.29)
HInRM2	66321680	33157547	0.0248	98.13	94.36	45.43	25915978 (78.16)
HInRM3	75333450	37663061	0.0246	98.18	94.49	45.43	29797145 (79.12)
HInS1	69084962	34540233	0.0247	98.17	94.46	46.03	27486151 (79.58)
HInS2	70023764	35010013	0.0248	98.14	94.36	45.71	27548357 (78.69)
HInS3	69682030	34838962	0.0244	98.26	94.7	46.55	27695194 (79.49)
LCpR1	47389730	23502486	0.0245	98.24	94.64	45.50	18306279 (77.89)
LCpR2	40607952	20172669	0.0243	98.34	94.83	45.23	15697425 (77.82)
LCpR3	48208192	23885427	0.0248	98.1	94.31	45.67	18606224 (77.9)
LCpS1	42392130	20994947	0.0243	98.3	94.81	45.63	16831601 (80.17)
LCpS2	47224364	23394629	0.0246	98.19	94.59	45.79	18375453 (78.55)
LCpS3	48991056	24280749	0.0245	98.25	94.67	46.33	18686304 (76.96)
LInM1	3822086	1910892	0.025	97.94	94.26	49.87	794889 (41.6)
LInM2	4222894	2111283	0.025	97.9	94.27	49.86	959150 (45.43)
LInM3	4000546	2000121	0.0251	97.88	94.22	49.87	913585 (45.68)
LInRM1	72572982	36284294	0.0241	98.46	94.92	45.60	27926438 (76.97)
LInRM2	57962844	28978389	0.0242	98.43	94.84	45.61	22017511 (75.98)
LInRM3	83811380	41901550	0.0242	98.44	94.86	45.65	32224240 (76.90)
LInS1	86880838	43438314	0.0242	98.44	94.88	46.09	33571165 (77.28)
LInS2	89127142	44558743	0.024	98.52	95.03	45.36	34577732 (77.60)
LInS3	82950854	41472309	0.0242	98.44	94.79	45.56	31757818 (76.58)

Table S3 Summary of transcriptomic functional annotations

	LS88		P. massoniana	
**Database**	Expression Gene number (rate %)	Expression Transcript number (rate %)	Expression Gene number (rate %)	Expression Transcript number (rate %)
GO	7625(69.29)	7625(69.29)	34004(42.22)	67597(48.37)
KEGG	4315(39.21)	4315(39.21)	15399(19.12)	31705(22.69)
EggNOG	1149(10.44)	1149(10.44)	30825(38.27)	62261(44.56)
NR	10890(98.96)	10890(98.96)	39385(48.90)	78305(56.04)
Swiss-Prot	6081(55.26)	6081(55.26)	28423(35.29)	57120(40.88)
Pfam	7421(67.44)	7421(67.44)	30982(38.47)	62182(44.50)
Total annotation	10894(99.00)	10894(99.00)	42222(52.42)	83452(59.72)
Total	11004(100)	11004(100)	80544(100)	139737(100)

Table S4 Transcriptomic submission information of LS88

Accession	Sample name	BioProject	SRA	Submission	BioSample organism name
SAMN38503235	HCfM3	PRJNA1041072	SRR27009363	SUB13987759	*Suillus luteus*
SAMN38503234	HCfM2	PRJNA1041072	SRR27009364	SUB13987759	*Suillus luteus*
SAMN38503225	HInM2	PRJNA1041072	SRR27009365	SUB13987759	*Suillus luteus*
SAMN38503224	HInM1	PRJNA1041072	SRR27009366	SUB13987759	*Suillus luteus*
SAMN38503232	HInS3	PRJNA1041072	SRR27009368	SUB13987759	*Suillus luteus*
SAMN38503233	HCfM1	PRJNA1041072	SRR27009367	SUB13987759	*Suillus luteus*
SAMN38503231	HInS2	PRJNA1041072	SRR27009369	SUB13987759	*Suillus luteus*
SAMN38503229	HInRM3	PRJNA1041072	SRR27009371	SUB13987759	*Suillus luteus*
SAMN38503230	HInS1	PRJNA1041072	SRR27009370	SUB13987759	*Suillus luteus*
SAMN38503228	HInRM2	PRJNA1041072	SRR27009372	SUB13987759	*Suillus luteus*
SAMN38503247	SLM3	PRJNA1041072	SRR27009374	SUB13987759	*Suillus luteus*
SAMN38503245	SLM1	PRJNA1041072	SRR27009376	SUB13987759	*Suillus luteus*
SAMN38503244	LInS3	PRJNA1041072	SRR27009377	SUB13987759	*Suillus luteus*
SAMN38503246	SLM2	PRJNA1041072	SRR27009375	SUB13987759	*Suillus luteus*
SAMN38503227	HInRM1	PRJNA1041072	SRR27009373	SUB13987759	*Suillus luteus*
SAMN38503226	HInM1	PRJNA1041072	SRR27009378	SUB13987759	*Suillus luteus*
SAMN38503241	LInRM3	PRJNA1041072	SRR27009381	SUB13987759	*Suillus luteus*
SAMN38503238	LInM3	PRJNA1041072	SRR27009384	SUB13987759	*Suillus luteus*
SAMN38503242	LInS1	PRJNA1041072	SRR27009380	SUB13987759	*Suillus luteus*
SAMN38503243	LInS2	PRJNA1041072	SRR27009379	SUB13987759	*Suillus luteus*
SAMN38503240	LInRM2	PRJNA1041072	SRR27009382	SUB13987759	*Suillus luteus*
SAMN38503239	LInRM1	PRJNA1041072	SRR27009383	SUB13987759	*Suillus luteus*
SAMN38503236	LInM1	PRJNA1041072	SRR27009386	SUB13987759	*Suillus luteus*
SAMN38503237	LInM2	PRJNA1041072	SRR27009385	SUB13987759	*Suillus luteus*

Note: Due to the presence of both *Suillus luteus* and *Pinus massoniana* transcriptomic information in certain samples, the corresponding transcriptomic data have been uploaded for both species to enable easy retrieval.

Table S5 Transcriptomic submission information of *P. massonian*

Accession	Sample name	BioProject	SRA	Submission	BioSample organism name
SAMN38274030	HCpR3	PRJNA1041072	SRR26882195	SUB13818680	*Pinus massoniana*
SAMN38274029	HCpR2	PRJNA1041072	SRR26882196	SUB13818680	*Pinus massoniana*
SAMN38274020	HInRM2	PRJNA1041072	SRR26882197	SUB13818680	*Pinus massoniana*
SAMN38274019	HInRM1	PRJNA1041072	SRR26882198	SUB13818680	*Pinus massoniana*
SAMN38274028	HCpR1	PRJNA1041072	SRR26882199	SUB13818680	*Pinus massoniana*
SAMN38274027	HCpS3	PRJNA1041072	SRR26882200	SUB13818680	*Pinus massoniana*
SAMN38274026	HCpS2	PRJNA1041072	SRR26882201	SUB13818680	*Pinus massoniana*
SAMN38274025	HCpS1	PRJNA1041072	SRR26882202	SUB13818680	*Pinus massoniana*
SAMN38274024	HInS3	PRJNA1041072	SRR26882203	SUB13818680	*Pinus massoniana*
SAMN38274023	HInS2	PRJNA1041072	SRR26882204	SUB13818680	*Pinus massoniana*
SAMN38274022	HInS1	PRJNA1041072	SRR26882205	SUB13818680	*Pinus massoniana*
SAMN38274042	LCpS3	PRJNA1041072	SRR26882206	SUB13818680	*Pinus massoniana*
SAMN38274041	LCpS2	PRJNA1041072	SRR26882207	SUB13818680	*Pinus massoniana*
SAMN38274039	LCpR3	PRJNA1041072	SRR26882209	SUB13818680	*Pinus massoniana*
SAMN38274040	LCpS1	PRJNA1041072	SRR26882208	SUB13818680	*Pinus massoniana*
SAMN38274021	HInRM3	PRJNA1041072	SRR26882210	SUB13818680	*Pinus massoniana*
SAMN38274038	LCpR2	PRJNA1041072	SRR26882211	SUB13818680	*Pinus massoniana*
SAMN38274037	LCpR1	PRJNA1041072	SRR26882212	SUB13818680	*Pinus massoniana*
SAMN38274036	LInS3	PRJNA1041072	SRR26882213	SUB13818680	*Pinus massoniana*
SAMN38274035	LInS2	PRJNA1041072	SRR26882214	SUB13818680	*Pinus massoniana*
SAMN38274034	LInS1	PRJNA1041072	SRR26882215	SUB13818680	*Pinus massoniana*
SAMN38274033	LInRM3	PRJNA1041072	SRR26882216	SUB13818680	*Pinus massoniana*
SAMN38274031	LInRM1	PRJNA1041072	SRR26882218	SUB13818680	*Pinus massoniana*
SAMN38274032	LInRM2	PRJNA1041072	SRR26882217	SUB13818680	*Pinus massoniana*

Note: Due to the presence of both *Suillus luteus* and *Pinus massoniana* transcriptomic information in certain samples, the corresponding transcriptomic data have been uploaded for both species to enable easy retrieval.

Table S6 Primers for potential key genes and TDF1

**Gene**		**sequence（5'-3'）**
TDF1	F	CCGGTTTCAGTAATACAGAGTCCT
	R	GGTCTGTTTACCTTACTTTATTATGTCCACC
evm.TU.Scaffold330.4 (ScaffoldD)	F	AAGAAACACCCCAACCAGGG
	R	CGATGGGCGTGACAAAAGTG
evm.TU.Scaffold2140.1 (ScaffoldB)	F	TCTCACTCACCTCTACCGCA
	R	GTTCCCAGCCATCTTCGACA

Table S7 Gene sequence alignment results on NCBI

Description	Max Score	Total Score	Query Cover	E value	Per. ident	Acc. Len	Accession
Suillus bovinus glutathione S-transferase (EDB93DRAFT_1252332), partial mRNA	233	233	26%	5.00E-56	88.21%	639	XM_041453278.1
Suillus subalutaceus glutathione S-transferase (DFJ58DRAFT_243473), mRNA	219	219	26%	1.00E-51	86.73%	863	XM_041379425.1
Suillus bovinus glutathione S-transferase (EDB93DRAFT_1329747), partial mRNA	211	211	26%	2.00E-49	86.29%	891	XM_041455150.1
Suillus paluster glutathione S-transferase (EDB91DRAFT_340398), mRNA	200	200	26%	5.00E-46	85.20%	1154	XM_041326926.1
Suillus subalutaceus glutathione S-transferase (DFJ58DRAFT_719538), partial mRNA	169	169	22%	1.00E-36	84.62%	633	XM_041384648.1
Suillus subaureus glutathione S-transferase (BJ212DRAFT_1446531), partial mRNA	147	275	38%	7.00E-30	82.25%	633	XM_041338263.1
Suillus subaureus glutathione S-transferase (BJ212DRAFT_1479843), partial mRNA	135	135	22%	2.00E-26	81.07%	567	XM_041339958.1
Suillus subalutaceus glutathione S-transferase (DFJ58DRAFT_918210), partial mRNA	132	238	37%	2.00E-25	80.84%	729	XM_041395586.1
Suillus subalutaceus glutathione S-transferase (DFJ58DRAFT_823604), mRNA	117	117	15%	5.00E-21	85.09%	838	XM_041392123.1

Table S8 Transcript sequence alignment results on NCBI

Description	Max Score	Total Score	Query Cover	E value	Per. ident	Acc. Len	Accession
Suillus subalutaceus glutathione S-transferase (DFJ58DRAFT_719538), partial mRNA	606	606	99%	2.00E-168	85.64%	633	XM_041384648.1
Suillus subaureus glutathione S-transferase (BJ212DRAFT_1446531), partial mRNA	601	601	99%	1.00E-166	85.47%	633	XM_041338263.1
Suillus bovinus glutathione S-transferase (EDB93DRAFT_1252332), partial mRNA	512	512	99%	5.00E-140	82.71%	639	XM_041453278.1
Suillus subalutaceus glutathione S-transferase (DFJ58DRAFT_243473), mRNA	501	501	99%	1.00E-136	82.45%	863	XM_041379425.1
Suillus discolor glutathione S-transferase (F5147DRAFT_773211), partial mRNA	501	501	99%	1.00E-136	82.38%	633	XM_041441428.1
Suillus fuscotomentosus glutathione S-transferase (F5891DRAFT_274015), mRNA	484	484	98%	1.00E-131	82.06%	1180	XM_041371158.1
Suillus plorans glutathione S-transferase (HD556DRAFT_1443114), partial mRNA	484	484	99%	1.00E-131	81.90%	633	XM_041306559.1
Suillus subalutaceus glutathione S-transferase (DFJ58DRAFT_823604), mRNA	479	479	90%	5.00E-130	83.30%	838	XM_041392123.1
Suillus subalutaceus glutathione S-transferase (DFJ58DRAFT_918210), partial mRNA	420	420	82%	3.00E-112	82.56%	729	XM_041395586.1
Suillus bovinus glutathione S-transferase (EDB93DRAFT_1329747), partial mRNA	418	418	99%	1.00E-111	79.90%	891	XM_041455150.1
Suillus paluster glutathione S-transferase (EDB91DRAFT_340398), mRNA	418	418	99%	1.00E-111	79.90%	1154	XM_041326926.1
Suillus subaureus glutathione S-transferase (BJ212DRAFT_1479843), partial mRNA	383	383	67%	4.00E-101	84.22%	567	XM_041339958.1
Suillus discolor glutathione S-transferase (F5147DRAFT_747365), partial mRNA	255	255	41%	9.00E-63	85.60%	573	XM_041439941.1

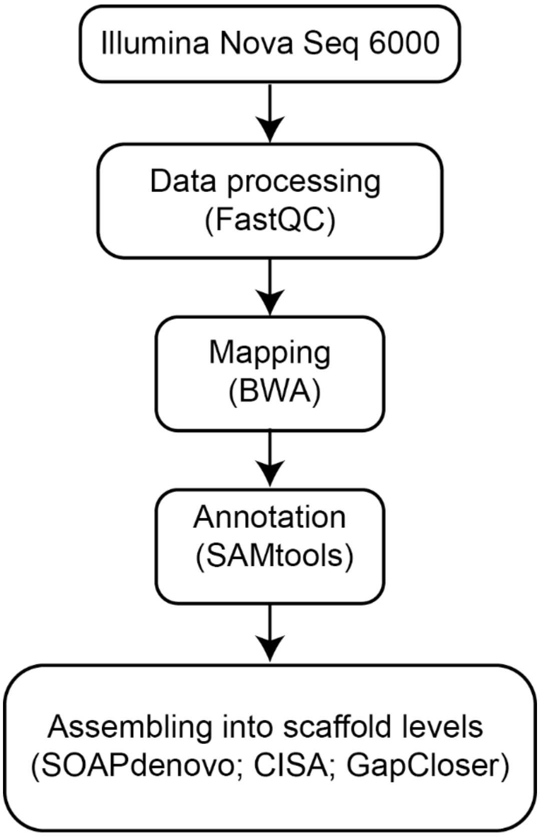

Fig. S1 Pipelines for LS88 genome resequencing analysis

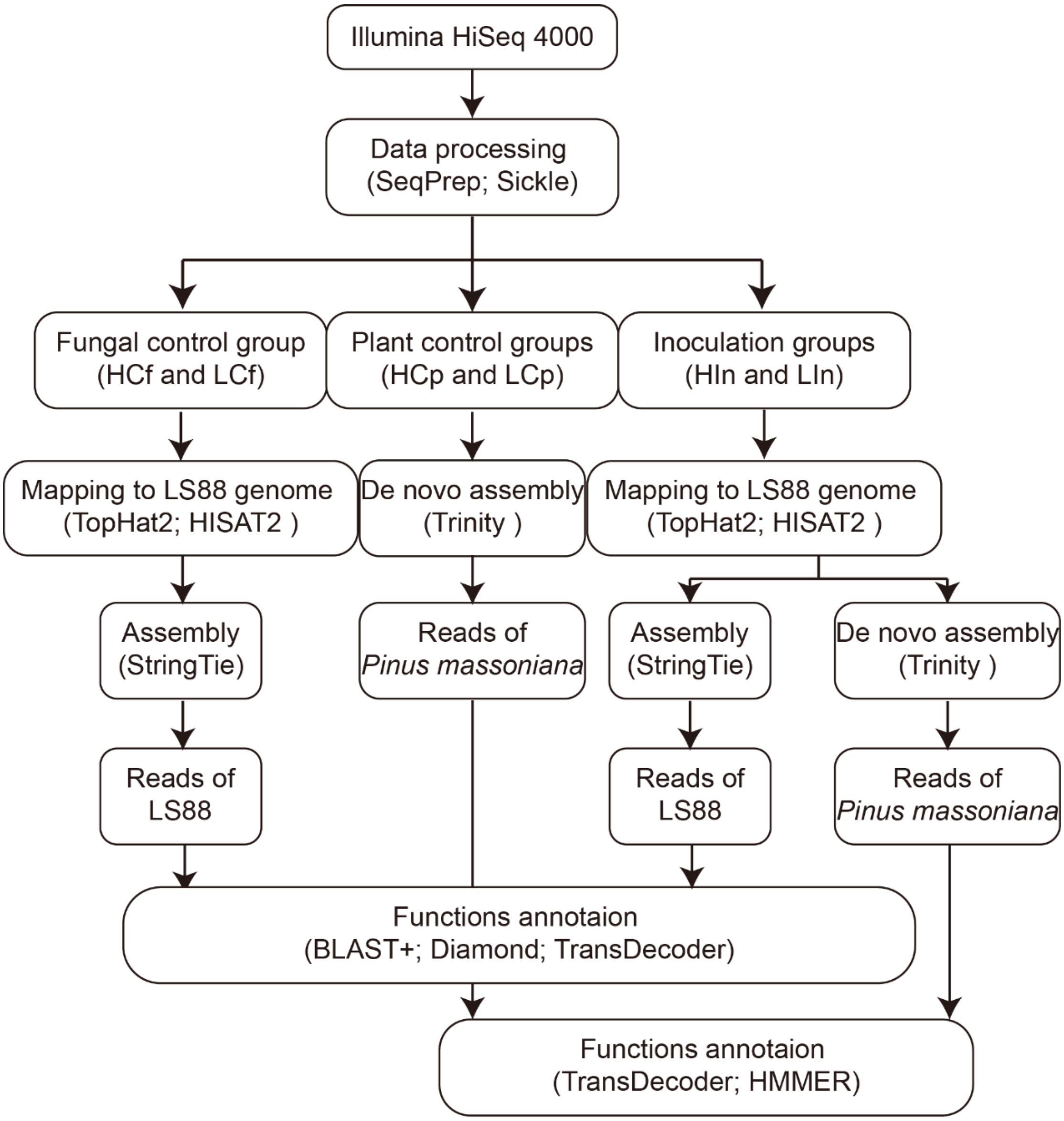

Fig. S2 Pipelines for transcriptome sequencing

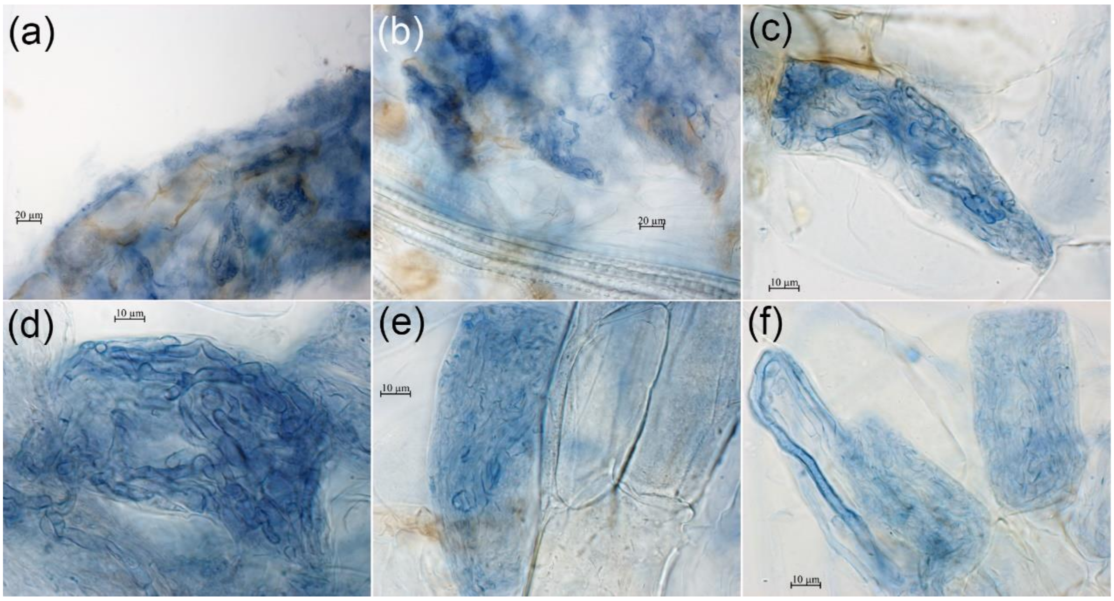

Fig. S3 Stained pictures of inoculated roots

Note: a) Mantles formed by hyphae in the root meristem zone; b) Hartig net covers root cortical cells without entering root vascular bundles; c) Root hair cells covered by Hartig net; d-f) Root cortical cells encased by Hartig net. a-b: The objective magnification is 50×, scale bar=20 µm; c-f: The objective magnification is 100×, scale bar=10 µm.

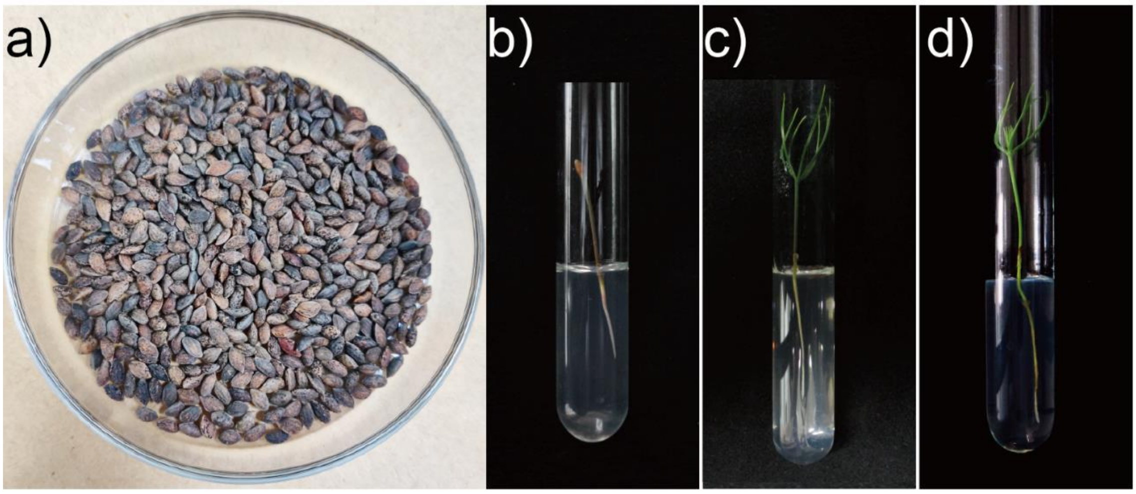

Fig. S4 Cultivation of sterile *P. massoniana* seedlings.

Note: a) Seeds chosen for germination treatment; b) Germination treatment for 10 days;c) Germination treatment for 20 days; d) Germination treatment for 30 days; the tube diameter was 18 mm. After 10 days (Fig. S4b), the seeds began to germinate, and the roots extended into the culture medium. After 20 days (Fig. S4c), some seedlings displayed fully expanded cotyledons. By the 30th day of treatment (Fig. S4d), sterile seedlings started developing true leaves, while the root system continued to elongate.
