# Supplementary figures and images for: Differential Strategies of Ectomycorrhizal Development between Suillus luteus and Pinus massoniana in Response to Nutrient Changes
Source: J Fungi (Basel). 2024 Aug 19;10(8):587. doi: 10.3390/jof10080587 (PMC11355094; doi:10.3390/jof10080587)

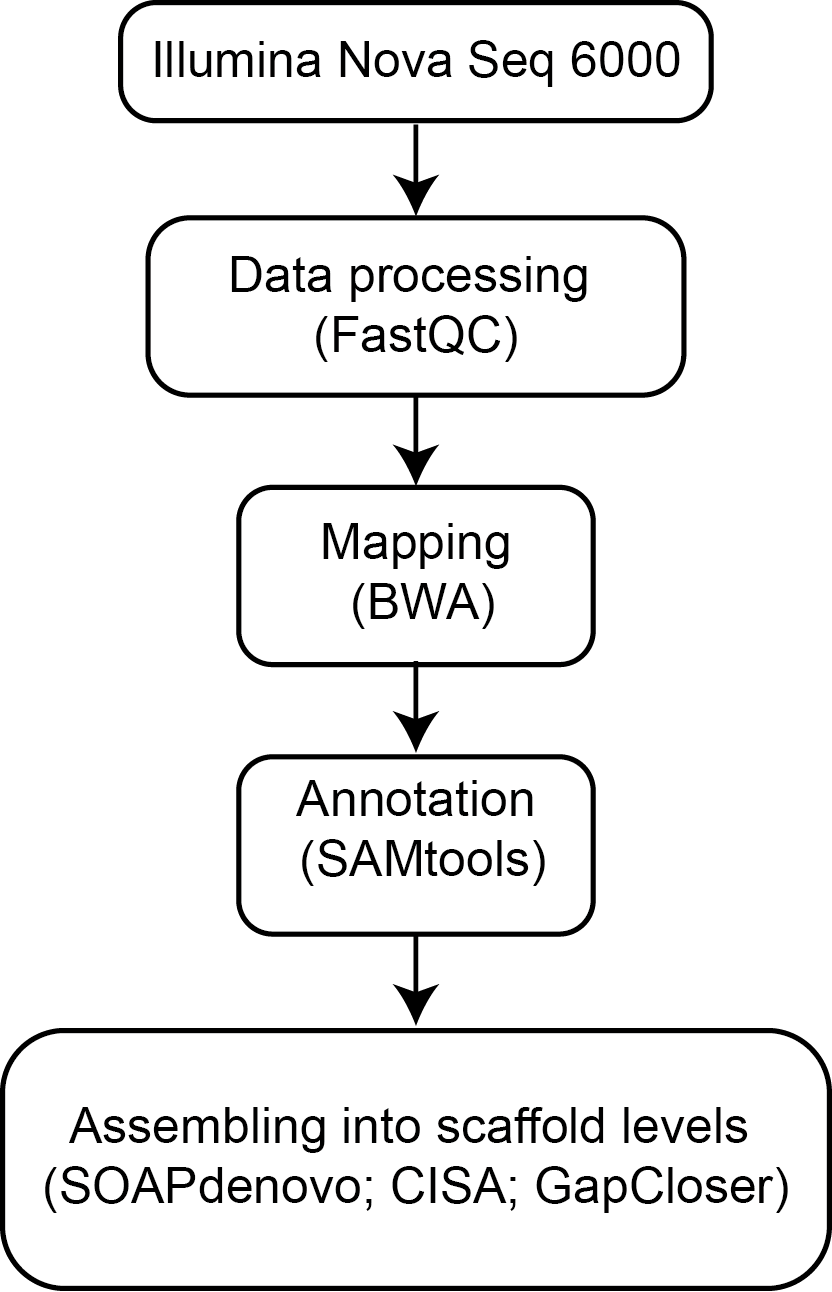

Supplement: Supplementary file 1 [file jof-10-00587-s001.zip › FigS1.tif]

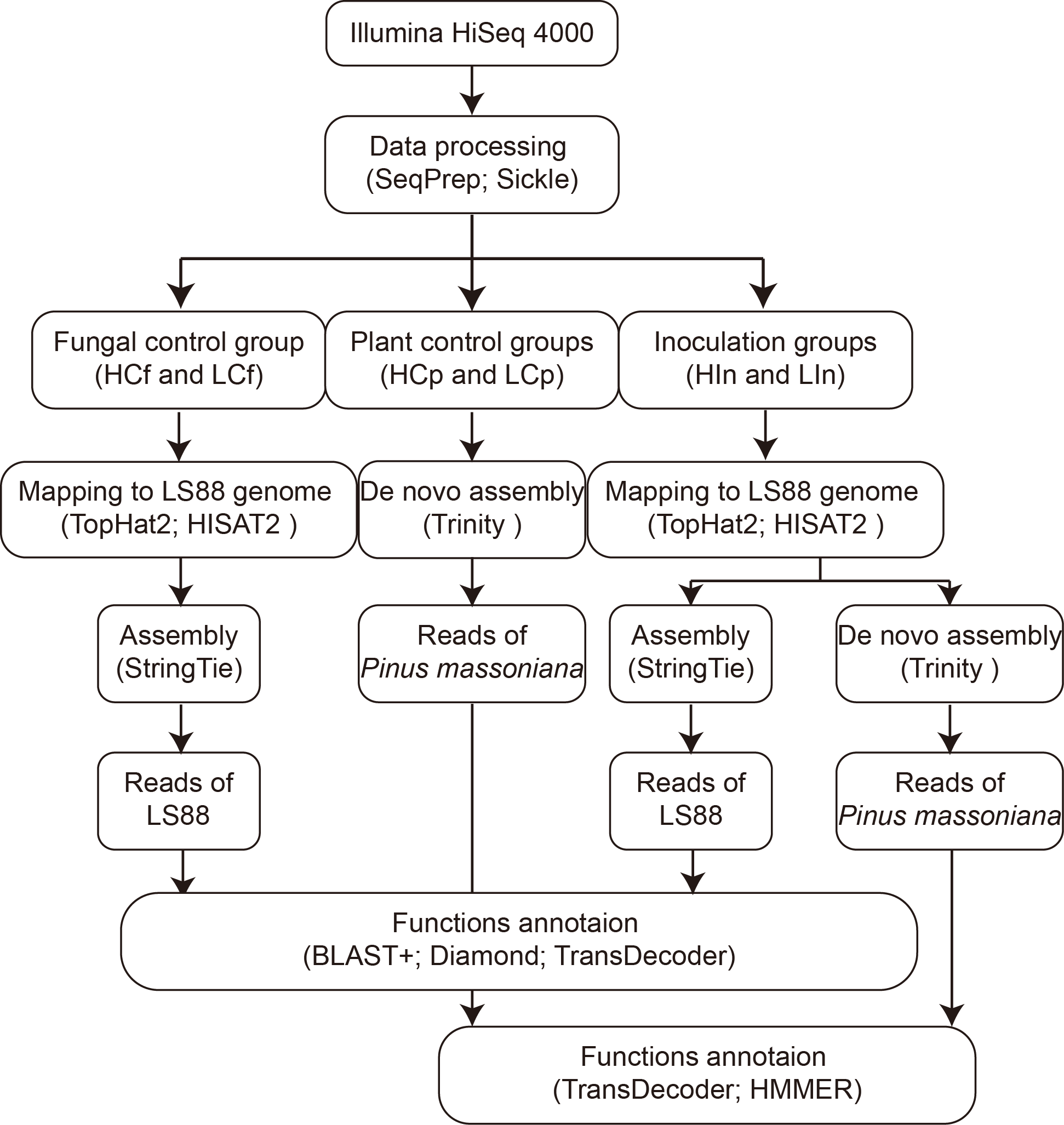

Supplement: Supplementary file 1 [file jof-10-00587-s001.zip › FigS2.tif]

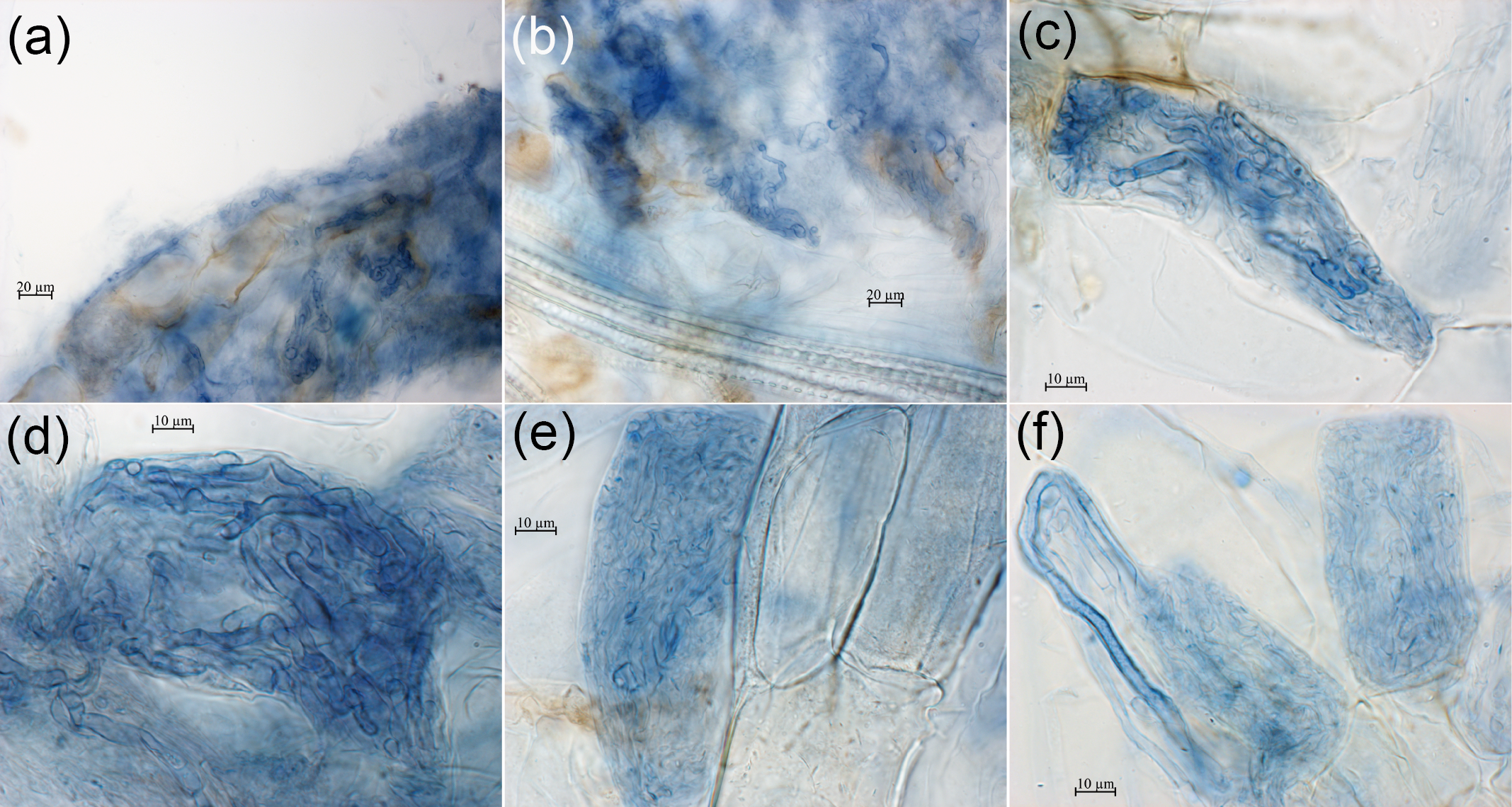

Supplement: Supplementary file 1 [file jof-10-00587-s001.zip › FigS3.tif]

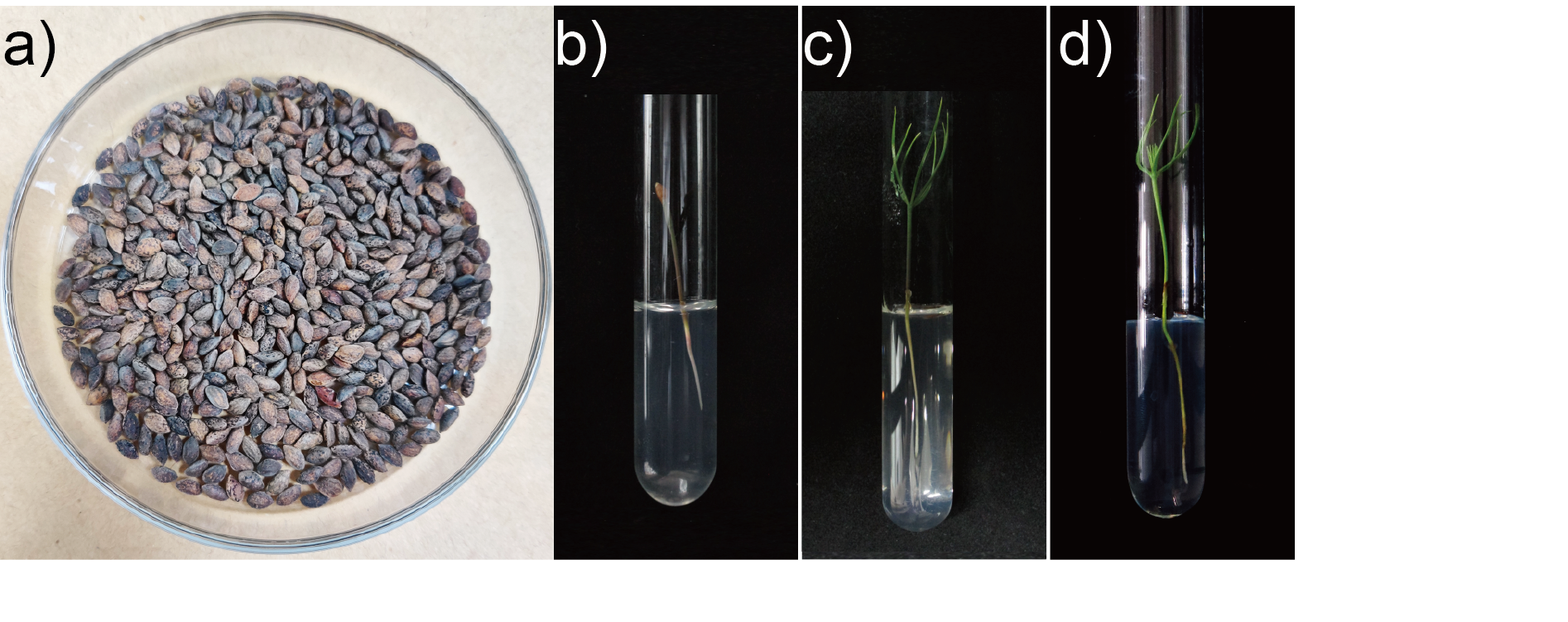

Supplement: Supplementary file 1 [file jof-10-00587-s001.zip › FigS4.tif]
